# Targeted Delivery of Protein Drugs by Nanocarriers

**DOI:** 10.3390/ma3031928

**Published:** 2010-03-17

**Authors:** Roberto Solaro, Federica Chiellini, Antonella Battisti

**Affiliations:** Department of Chemistry and Industrial Chemistry, University of Pisa, Via Risorgimento 35, 56126, Pisa, Italy; E-Mails: federica@dcci.unipi.it (F.C.); antobattisti@dcci.unipi.it (A.B.)

**Keywords:** nanocarrier, targeted delivery, protein drug

## Abstract

Recent advances in biotechnology demonstrate that peptides and proteins are the basis of a new generation of drugs. However, the transportation of protein drugs in the body is limited by their high molecular weight, which prevents the crossing of tissue barriers, and by their short lifetime due to immuno response and enzymatic degradation. Moreover, the ability to selectively deliver drugs to target organs, tissues or cells is a major challenge in the treatment of several human diseases, including cancer. Indeed, targeted delivery can be much more efficient than systemic application, while improving bioavailability and limiting undesirable side effects. This review describes how the use of targeted nanocarriers such as nanoparticles and liposomes can improve the pharmacokinetic properties of protein drugs, thus increasing their safety and maximizing the therapeutic effect.

1. Introduction................................................................................................19292. Challenges and limitations for the delivery of protein drugs.................19303. Nanocarriers...............................................................................................1931 3.1. Liposomes...........................................................................................1933 3.2. Virosomes...........................................................................................1936 3.3. Solid Lipid Nanoparticles..................................................................1937 3.4. Polymeric Nanoparticles...................................................................1940 3.5. Protein Conjugates.............................................................................19484. Targeting strategies and applications.......................................................1950 4.1. Topical Application............................................................................1950 4.2. Enhanced Permeability and Retention Effect..................................1951 4.3. Physical Targeting..............................................................................1953 4.4. Molecular Targeting...........................................................................19565. Concluding remarks..................................................................................1961Acknowledgements.......................................................................................1962References.....................................................................................................1962

## 1. Introduction

Recent advances in biotechnology allow the selection and the preparation of novel macromolecular compounds such as peptides, proteins and DNA analogs to be used as drugs (e.g., hormones, monoclonal antibodies, vaccines) for therapeutic purposes. Such compounds show powerful and selective therapeutic activity, but unfortunately they must often be dropped at some development stage, because of their high enzymatic susceptibility, short shelf life or unsuitable efficacy after the administration to the patient, owing to immunogenic reactions or poor bioavailability [1]. In some cases, from a physicochemical point of view, they cannot reach or enter target cells. Moreover, the drug must cross several biological barriers to reach the site of action, and along its path it can be inactivated or produce undesired side effects. Several approaches have been evaluated to overcome these issues. The first one simply consists of applying the drug to the affected area, since this method minimizes undesired side effects following systemic administration. However, direct application cannot overcome problems connected to the nature of protein drugs, which are hydrophilic and have a large size and an intrinsic instability due to denaturation processes. They also are rapidly filtered by the kidneys or intercepted by the immune system.

Drug targeting is a promising tool to solve most of the aforementioned problems. This approach consists of designing a system able to selectively deliver the drug to the area of interest. Transport systems can be designed to control the dispatch of the loaded drug to target areas, increasing its local concentration and bioavailability, while prolonging its retention, half-life and effectiveness. This strategy can avoid diffusion of the drug into normal organs, thus avoiding negative side effects. It is outstanding how favorable this method can be: it can improve pharmacokinetics [2]; it works independently of the administration method; it minimizes the required amount of drug and hence the cost of the therapy. Over a hundred years ago, Paul Ehrlich was the first one to theorize the use of a “magic bullet” to deliver drugs within the body [3]. His idea consisted of the use of an entity able to selectively recognize the pathological agent (cells, bacteria or other microorganisms) and to destroy it. The targeting process should assure that the pharmacological effect could only be expressed in the targeted area. Nowadays, these systems have evolved in a structure composed of three major blocks: the pharmacologically active substance, a carrier used to increase the number of active molecules per system (frequently a nanosized carrier) and a targeting moiety able to lead the whole system to the selected site of action. In a branch of his extensive work, Ehrlich identified antibodies as the best targeting moieties, owing to their high affinity and specificity for the relevant antigen. Since then, several different targeting techniques have been investigated, and in parallel many different kinds of carrier have been developed, according to novel information about toxicity, tolerability, biocompatibility and acceptability of properly designed materials by living organisms.

This review gives a survey of the different nanocarriers and targeting strategies employed for the specific delivery of pharmaceuticals, with a special focus on peptide and protein based drugs.

## 2. Challenges and Limitations for the Delivery of Protein Drugs

The physicochemical properties are the main factor that influence the diffusion of a drug within the body. In particular, protein drugs show high hydrophilicity, large size and substantial physical and chemical lability; these features strongly influence the pharmacokinetic and pharmacodynamic behavior of the drug *in vivo*. They also limit the reactions, solvents and environmental conditions that can be used in the preparation and application of protein- or peptide-based pharmaceuticals. Most of the commonly employed protein drugs are administered systemically by intramuscular, intravenous, subcutaneous and intraperitoneal injections, and formulations often include excipients (e.g., buffers, preservatives, solubility enhancers), whose major role is that of improving the *in vivo* stability of the biomolecule. In this respect, surfactants and albumin play an important role in reducing aggregation and the adsorption processes, thus limiting the possibility of protein unfolding, deactivation or precipitation [4].

High molecular weight, hydrophilicity, structural fragility, and complexity are the main obstacles to the use of protein drugs [5,6]. Indeed, these macromolecules can easily undergo degradation, denaturation and eventually inactivation by physical, chemical, and enzymatic mechanisms during formulation, storage, and delivery. Additionally, they have poor biopharmaceutical properties [7]. The degradation by the proteolytic enzymes located in the gut, lungs, and skin, and the poor mucosa permeability strongly limit protein bioavailability [8]. One of the most important problems to the therapeutic performance of protein drugs is due to the rapid clearance from the body owing to glomerular filtration, endocytosis, phagocytosis, enzyme degradation, and immunosystem processing [9]. Xeno-proteins are intrinsically immunogenic and antigenic. Small proteins are mainly excreted by the kidneys, whereas large proteins usually undergo enzyme degradation. Lipoproteins and glycosylated proteins are selectively taken-up by endocytosis or phagocytosis by the reticuloendothelial system (RES) [10]. Often, hormones and cytokines are eliminated from circulation by receptor-mediated endocytosis and intracellular processing [11]. Moreover, most physiological proteins are synthesized at local sites without reaching appreciable systemic levels. Thus far, attempts to improve the protein bioavailability and targeting have ranged from tailoring the physicochemical properties of peptide molecules to the inclusion of functional excipients into specially adapted drug delivery systems.

According to the “binding site barrier” theory [12], ligands with very high affinity for their targets will bind extremely tightly to the binding sites immediately adjacent to the blood vessel. This creates a physical barrier for subsequent drug molecules and causes incomplete drug penetration. The effect of binding site barrier also depends on the density of targeted molecules on cell surface and the turnover rate of target molecules. Obviously, the binding site barrier is a serious concern for high-affinity monoclonal antibodies. Increasing dose, lowering affinity, and decreasing ligand size can however improve target tissue penetration.

As proteins reach the surface of target cells, the plasma membrane constitutes the first substantial hurdle for cellular uptake of protein therapeutics. Indeed, most protein drugs require efficient intracellular delivery to exert their therapeutic effects. The intracellular organization of mammalian cells is highly complex with extensive compartmentalization that imposes additional barriers for protein drugs that need to reach intracellular targets.

As indicated, effective use of protein drugs can be compromised by their instability in the body, rapid rates of clearance, premature uptake by tissues and immunogenicity or antigenicity [13]. Conjugation to poly(ethylene glycol) (PEG) chains - that is PEGylation [14] - endows protein and peptide drugs with longer circulatory half-lives and reduced immunogenicity. An increasing number of PEGylated drugs are now used clinically (e.g., asparaginase, interferon α, tumor necrosis factor and granulocyte-colony stimulating factor) [13]. However, PEGylated proteins can generate anti-PEG antibodies that could influence the residence time of the conjugate in the circulating blood. So far, no adverse effects of PEG immunogenicity have been observed, possibly because of the very small amounts of injected PEGylated drugs currently in use [15].

## 3. Nanocarriers

The term “nanoparticle” is broadly applied in the description of almost every pharmaceutical carrier or imaging agent system, so further classification is needed for clarity [16]. One group of nanocarriers includes single-chain polymer–drug conjugates, polymer colloids prepared by techniques such as emulsion polymerization, crosslinked nanogel matrices, dendrimers, and carbon nanotubes. For this group, the carrier is a single synthetic molecule with covalent bonds and a relatively large molar mass. Other types of nanocarriers, often termed nanoparticles, comprise self-assemblies of smaller molecules, which are aggregated through intermolecular forces. Liposomes and polyplexes are the most studied members of this class of particles, but this class of carriers also includes aggregates such as polymersomes and other assemblies of block copolymers, colloidosomal aggregates of latex particles, and protein or peptide assemblies. The dynamic nature of these types of systems depends upon the intermolecular forces in play and the biological conditions. Finally, nanocarriers include also complexes based upon fullerenes, silica, colloidal gold, gold nanoshells, quantum dots, and superparamagnetic particles.

The use of a properly designed carrier for the sustained and targeted delivery of pharmaceuticals offers several advantages compared with classic administration: it can increase the amount of drug that reaches the targeted area, improve the transportation mechanism and protect the drug against inactivation, degradation and metabolization phenomena. The main characteristics that the carrier must show are:

■ The ability to encapsulate the drug without deactivating it;
■ The possibility for releasing the drug under proper conditions and according to proper kinetics;
■ A high stability and long circulation time after administration;
■ The capability to actively or passively deliver the drug to a target area.

Above all, the use of nanosized carriers offers a way to cross biological barriers that would otherwise forbid the drug to accede to the site of interest, as it often happens in the central nervous system or in the gastrointestinal tract. Nanocarriers have a high surface area to volume ratio, thus providing improved pharmacokinetics and biodistribution of drugs while minimizing toxicity, thanks to specific targeted transport [17]. Moreover, they can improve the solubility of many drugs and prolong the shelf-life and *in vivo* stability of peptides, proteins and oligonucleotides [18,19]. In particular, the use of biodegradable materials, which has already been reviewed [20,21,22], minimizes the risk for hypersensitivity reactions and ensures good tissue compatibility [23]. Among the potential nanocarriers, colloidal systems such as liposomes [24,25,26,27,28,29,30,31] and nanoparticles [29,30,32,33,34,35,36,37,38,39] have aroused considerable interest and have been extensively reviewed.

Complex drug delivery systems are thus a potential alternative to the conventional formulations of proteins, in which the protein is usually either lyophilized, in suspension, or in an aqueous solution. The optimal release pattern may vary between proteins and between indications, and adaptable formulations are therefore required. Some proteins require sustained release, while others require controlled, immediate or pulsed release. Release can be obtained with different particulate drug delivery systems [5]. Liposomes, solid-lipid nanoparticles, polymeric nanoparticles and virosomes are the most commonly used nanocarriers for protein delivery.

In many cases, targeted or untargeted liposomes and nanoparticles are rapidly cleared from the blood stream by the RES; although this event is usually considered a disadvantage, it can lead to the aim of activating macrophages if required by certain therapies. Since macrophages are mostly located in the spleen and liver, their ability to catch particles can be used to selectively deliver substances to these organs. Otherwise, specific chemical modification of the carrier can be performed in order to make the system able to avoid the RES. Surface modification of nanocarriers is commonly performed to give them suitable biological properties, to prolong their life in the blood stream, to limit the uptake by macrophages, and to make them able to target specific organs or tissues.

Nanoparticles, such as liposomes, polymeric micelles, lipoplexes and polyplexes have been extensively studied as targeted drug carrier systems over the past three decades. A wide variety of active agents can be incorporated into or complexed with these particles, varying from low molecular weight drug molecules to macromolecules such as proteins and nucleic acids. An important requirement to the systemic intravenous use of this targeted nanomedicine approach is the ability of the nanoparticles to circulate in the bloodstream for a prolonged period of time. To achieve this, PEG is often used as a coating material. It is generally assumed that PEG creates a so-called “steric stabilization” effect: the PEG molecules form a protective hydrophilic layer on the surface of the nanoparticle that opposes interaction with blood components. As a result, the PEG coating reduces uptake by macrophages of the mononuclear phagocyte system (MPS) and provides relatively long plasma residence times [40]. Until now, PEG is still the most widely used material for achieving steric stabilization. Nevertheless, successful attempts have been made to design alternative polymers, for example polymers based on polyoxazoline, poly(vinyl alcohol), polyglycerol, poly(N-vinyl-2-pyrrolidinone), poly(*N*-(2-hydroxypropyl)methacrylamide) and poly(amino acid)s [41]. For all these coating materials, prolonged circulation times of nanoparticles as compared to non-coated nanoparticles have been reported.

It is generally assumed that the macrophage-resistant property of sterically protected particles is due to suppression in surface opsonization (protein adsorption facilitating uptake) and protein adsorption. However, recent evidence shows that sterically stabilized particles are prone to opsonization, particularly by the opsonic components of the complement system. Moghimi and Szebeni [42] evaluated these phenomena and discussed theories that reconcile complement activation and opsonization with prolonged circulation times. With respect to particle longevity, the physiological state of macrophages also plays a critical role. For example, stimulated or newly recruited macrophages can recognize and rapidly internalize sterically protected nanoparticles by opsonic-independent mechanisms.

Moreover, steric stabilization is not desirable for all steps in the drug targeting process. The prolonged circulation time is needed to enable extravasation at sites with increased vascular permeability such as tumors and inflamed sites (enhanced permeability and retention (EPR) effect) [43]. However, after localizing to the pathological site, nanoparticles should deliver their contents in an efficient manner to achieve a sufficient therapeutic response. The polymer coating may hinder drug release and target cell interaction and can therefore be an obstacle in the realization of the therapeutic response [44]. Attempts have been made to enhance the therapeutic efficacy of sterically stabilized nanoparticles by means of shedding, *i.e.,* loss of the coating after arrival at the target site [41]. This “unmasking” process may facilitate drug release and/or target cell interaction processes. The shedding concept provides a potential solution for several situations in which the polymer coating can have a negative effect on the delivery process.

Systemically administered nanomedicines should have diameters ranging from 10–200 nm: nanocarriers must be larger than 10 nm to avoid first-pass elimination through the kidneys and smaller than 200 nm to avoid sequestration by the spleen and liver [45]. The size of the fenestrations in tumor vasculature also puts an upper limit on the size of nanocarriers that exploit the EPR effect to accumulate in tumors. Tumor vasculature fenestrations vary depending on a myriad of factors, but usually do not exceed several hundred nanometers. Nanocarrier size has been shown to influence circulation half-life and tissue accumulation [46]. Size is not the only important property of drug nanocarriers. Surface properties can increase nanoparticle stability, prolong circulation in the blood [47] and dramatically influence opsonization and uptake by the RES. The route of administration can also affect nanoparticle biodistribution and targeting. The preferred route of administration for nanocarriers is often by intravenous injection. Other routes of administration have generally proven less efficacious, usually because less drug reaches the target tissues. On the other hand, subcutaneous or intradermal injections may be preferable for lymphatic targeting [48], whereas intraperitoneal injection may be more efficacious for brain targeting [49].

### 3.1. Liposomes

**Figure 1:**
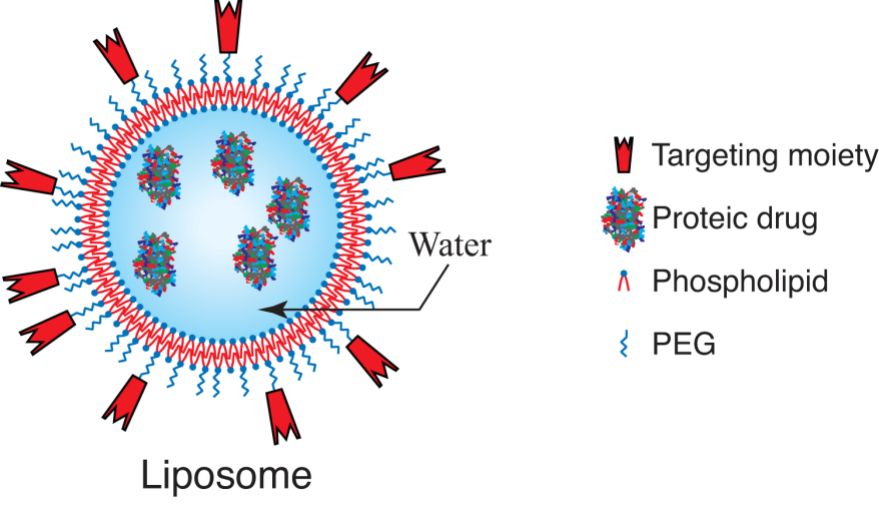
Structure of a unilamellar liposome for drug delivery.

Liposomes are micro or nanometric vesicles composed of amphiphilic species, such as lipids or phospholipids, that spontaneously form one (unilamellar) or more (multilamellar) concentric bilayers separated by water compartments (Figure 1). Lipids expose their hydrophilic head outwards, while the hydrophobic tail is directed inwards in the bilayer. Depending on the number of bilayers, the particle size can range from about 20 nm to several micrometers.

Liposomes show very versatile properties in terms of size, surface charge and lipid composition, and their ability to incorporate almost any drug independent of its solubility in water makes these microreservoir systems useful for delivery purposes. There is a large variety of lipids employable for the preparation of liposomes, comprising for instance mixtures of stearic acid and Tween 80 [50], mixtures of distearoyl phosphatidylcholine, distearoyl phosphatidylglycerol, and cholesterol [51], diplasmenylcholine [27], mixtures of distearoyl phosphatidylcholine, dimyristoyl phosphatidyl­glycerol, and cholesterol [52], mixtures of castor oil, phosphatidylcholine and polyethylene glycol coupled to distearoylphosphatidylethanolamine [53]. The stability of protein-based drugs can be maintained using adequate incorporation processes such as reverse-phase evaporation, injection and freeze-thaw. Liposomes also proved to be safe *in vivo* [54] and were even tested as possible candidates to improve the safety in prescribing drugs during pregnancy [55].

Liposomes can be exploited for drug delivery *via* pulmonary route. This method exhibits numerous benefits as an alternative for repeated injection of drugs like insulin. Encapsulation of insulin into liposomal carriers for pulmonary delivery (dry powder inhalation) showed good hypoglycemic effect with low blood glucose level and long-lasting period and a relatively high pharmacological bioavailability [56].

Liposomes are suitable carriers for the delivery of pro-apoptotic membrane proteins into cancer cells to induce cell death. Voltage-dependent anionic channel (VDAC) and Bak are two mitochondrial outer membrane proteins involved in the activation of the intrinsic apoptotic pathway that have been successfully conjugated to liposomes and delivered into HCT116p53^+/+^ and HCT116p53^−/−^ cells [57].

It is well known that the use of liposomes as suitable carriers for drug delivery is prone to issues connected with the phagocytic activity of macrophages [58]. The clearance of liposomes from the blood stream depends not only on mechanical filtration and membrane fusion events, but also on interactions with serum proteins and cellular receptors. Cellular receptors do not directly recognize the liposome as a foreign body, but they recognize specific serum proteins that bind the liposome surface (opsonins) [59]. Once the liposome has been internalized into a cell by endocytosis, several strategies can be used to achieve endosomal escape of liposome-encapsulated drugs. A recent technique consists of providing the cell with photosensitizer molecules, which primarily accumulate in endosomal membranes, and then exposing the system to light. Upon illumination, reactive singlet oxygen species form and damage the endosomal membrane, which becomes permeable to endocyted molecules (photochemical internalization). This method was successfully used to induce cytotoxicity in EGF-receptor positive human ovarian cancer cells, thanks to the plant toxin saporin encapsulated into targeted liposomes [60].

Liposomes can also undergo lipid exchange with high-density lipoproteins in blood, leading to liposome disintegration [61]. The intrinsic instability of liposomes in the body environment causes fast release of the loaded drug. This means a peak in drug concentration a short time after administration (burst effect). Liposomes can be protected against the burst effect, e.g., by encapsulation in alginate shells crosslinked with Ba^2+^ ions [62]. They can also be properly designed in order to minimize the macrophage uptake and prolong their circulation time after intravenous injection. The most exploited modification used to reach this goal is PEGylation. When the linear, non-toxic, flexible and hydrophilic PEG polymer that shows anti-opsonizing properties [63,64,65] is grafted on the liposome surface, it forms a hydrophilic layer that shields the liposome surface charge. Its steric hindrance and hydrophilicity prevent opsonins from settling on the liposome surface, making it difficult for RES cells to recognize and interact with liposomes. For this reason, such liposomes are commonly named stealth liposomes, long-circulating liposomes or sterically stabilized liposomes. It has been reported that free PEG and bound PEG produce opposite effects: free PEG causes fusion of the particles, while bound PEG protects them [66]. PEG-functionalized lipids exhibit dose-independent pharmacokinetics in animals and humans, and the ability to cross biological barriers *in vivo* [67,68]. These features allow PEGylated liposomes improved delivery and therapeutic efficacy of anti-cancer drugs [69,70]. An important aspect to be considered in liposome PEGylation is the choice of the PEG-lipid conjugate alkyl chain, which can in some cases produce immunogenicity after repeated administration [71].

Woodle *et al.* [72] investigated the value of novel systemically long-circulating liposomes to prolong the duration of an antidiuretic hormone, arg8-vasopressin (VP), as a representative of low molecular weight peptides with rapid clearance. The cholesterol content was found to have a controlling effect on VP release in serum. Three types of liposomes were tested in VP-deficient Brattleboro rats. One contained phosphatidylserine (PS), which was rapidly cleared from the circulation. In the other two liposomes, PS was replaced by either phosphatidylglycerol or a novel phospholipid derivatized with polyethylene glycol (PE-PEG); both showing prolonged circulation. The duration of the prolonged bioactivity was not dose dependent, but the amplitude was. This is attributed to VP release from liposomes, which were distributed intact to another compartment without being taken up by the RES. The authors concluded that liposomes could be applied to prolong the biological activity of a therapeutic peptide by balancing liposome circulation time, release rate, and dose.

Kedar *et al.* [73] demonstrated that recombinant human interleukin-2 (IL-2) can be successfully encapsulated in unilamellar, long-circulating, sterically stabilized liposomes. They also compared the immunomodulatory and anti-tumor effects of IL-2, pegylated IL-2 (PEG-IL-2) and liposome encapsulated IL-2 (SSL-IL-2) in mice. They found that SSL-IL-2 was significantly more effective than IL-2 in increasing leukocyte number in the blood and spleen and triggering spleen lymphokine-activated killer cell activity. The survival of mice with advanced metastatic carcinoma (previously treated with cyclophosphamide chemotherapy) was two to six times greater following administration of SSL-IL-2 than IL-2. Moreover, successful treatment with SSL-IL-2 required lower cumulative doses and fewer administrations. PEG-IL-2 was a more potent immunostimulator than SSL-IL-2 in normal mice, and as effective as SSL-IL-2 in tumor-bearing mice. PEG-IL-2, however, caused marked toxicity, including severe thrombocytopenia.

PEGylation is also frequently used to prepare radiolabeled liposomes for imaging techniques. PEGylated liposomes labeled with ^111^In administered intravenously to rats affected by *Staphylococcus aureus* showed that their clearance from the blood stream is similar to that of control ^111^In-IgG. On the contrary, the uptake by the inflammatory site was twice that of the control, making the inflammation visible by scintigraphy one hour after injection [74].

However, PEGylation is not the only modification reaction that can be carried out on liposomes. To improve the efficacy of ligand binding to a liposome membrane, Yagi *et al.* developed a novel lipid analog based on amino acids for liposome modification [75]. This lipid consists of three peptide derivatives and two fatty acids, and it was used to prepare liposomes incorporating the HIV-TAT peptide (domain of human immunodeficiency virus TAT protein). This is a protein transduction domain commonly employed to investigate the delivery of macromolecules, nucleic acids and liposomes into cells. Liposomes containing the lipid analog bearing HIV-TAT peptide exhibited efficient cellular uptake.

Other peptides can be used to modify liposomes surface. Octaarginine oligopeptide (R8) bound on the surface of liposomes can enhance cell internalization by macropinocytosis. R8-modified liposomes can escape from macropinosomes into the cytosol, preserving the encapsulated drug from degradation. Green fluorescence protein was chosen as a model protein and efficiently delivered into mitochondria thanks to highly mitochondrion-fusogenic lipid formulation of the liposomes [76].

Lipid modification can also alter the drug-loading efficacy by inducing changes in the membrane properties, such as micropolarity, microviscosity and free volume. Incorporation of cholesterol proved to reduce the partitioning of porphyrins, while methyl oleate and PEGylated lipids noticeably increased the value of the relevant binding constants [77]. Another important feature deriving from lipid modification is the insertion of functional groups able to bind ligands to the surface of liposomes. Ligands can react with the functional groups either before or after the liposome formation.

### 3.2. Virosomes

**Figure 2:**
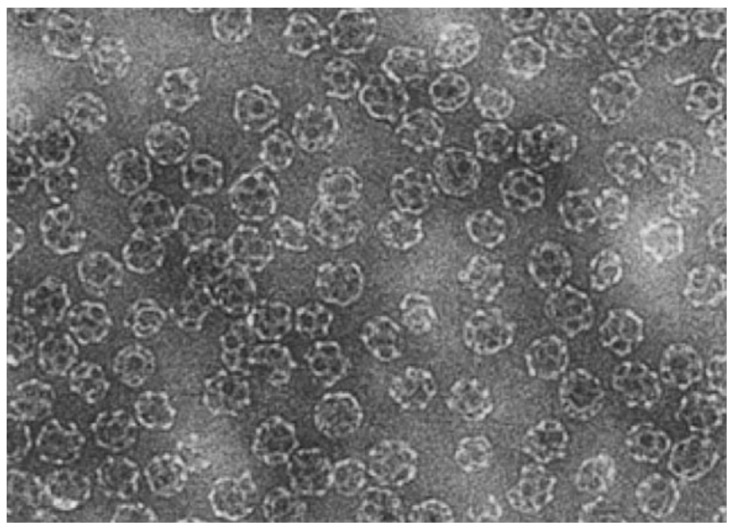
Electron micrograph of immune stimulating complexes showing 40 nm particles containing envelope proteins from influenza virus. Cholesterol and Quillaja saponin form hexagonal rings glued together by lipid, e.g., phosphatidylcholine, to form the spherical structure. Reprinted with permission from ref. [85], Copyright © 2004, Elsevier B.V.

The natural ability of viruses to enter and infect specific cell types can be exploited to deliver drugs into the cytosol [78,79,80]. Indeed, the virus shell has the ability to bind to target cell receptors and to fuse with the membrane. Clearly, to use this kind of nanocarrier for drug delivery, the viral genetic information must be removed from the virus shell in order to avoid infection of the cell. The emptied viral shell (virosome) can be used to deliver molecules (e.g., DNA [81], RNA [82], antigens [83], vaccines [84,85]) directly into cells, especially in gene therapy (Figure 2). Virosomes are often obtained from influenza virus by solubilization of the viral membrane followed by ultracentrifugation and reconstitution of the envelope by elimination of the detergent [86]. Since virus derivatives often show high immunogenic properties, fusogenic viral envelope proteins [87] or their synthetic analogs [88,89] can be combined with liposomes to obtain fusogenic capacities [90,91] while minimizing the immune response. The drug loading technique can also affect the delivery efficiency. Plasmid DNA can be delivered to target cells thanks to reconstituted influenza virosomes with good results *in vitro*, while *in vivo* the virosome-associated DNA is rapidly degraded by nuclease enzymes. The use of dicaproylphosphatidylcholine for solubilization of the viral membrane prevents its degradation by nucleases, thus making the DNA-virosome suitable for *in vivo* use [92].

### 3.3. Solid Lipid Nanoparticles

Solid lipid nanoparticles (SLNs) were first described in the nineties [93]. They are made of solid lipids well tolerated by the body (e.g., glycerides composed of fatty acids, which are commonly used in emulsions for parenteral nutrition, cholesterol [94], glycerol behenate (Compritol^®^ 888 ATO) [94], glyceryl palmitostearate (Precirol^®^ ATO 5) [95], glyceryl monostearates (Imwitor^®^ 900) [96], tripalmitin [97] and other triglycerides such as tristearin, trilaurin, hard fats such as Witepsol series, cetyl palmitate, lipid acids such as stearic acid [98], palmitic acid [99]), thus minimizing the risk of acute and chronic toxicity [100]. SLNs are solid at room temperature, thus allowing reduced mobility for incorporated drugs, which is a desirable feature for controlled drug release. Their diameter usually varies between 50 nm and 1 µm, and they can be stabilized using non-toxic surfactants, polymers or both. Large-scale production can be performed in a cost-effective and relatively simple way using hot or cold high-pressure homogenization (HPH) or microemulsion techniques [98]. Other possible preparation methods, such as emulsification-solvent evaporation [101], solvent injection [102], solvent emulsification-diffusion [103,104] and ultrasonication [105], require the use of organic solvents and do not allow for easy scale up.

Among particulate formulations, solid lipid nanoparticles have been successfully explored for drug delivery because they combine the benefits of liquid lipid-based colloidal systems (e.g., emulsions and liposomes) and solid systems [106]. These products possess excellent tissue biocompatibility, biodegradability, composition flexibility and small size, making them suitable for a variety of applications. Furthermore, they have been found to enhance the drug bioavailability after oral or local administration. On the other hand, solid lipid particle manufacturing techniques are not easily adaptable to protein processing as they operate under high temperature, pressure, and shear stress, which are detrimental to protein stability. To overcome these issues, techniques based on supercritical fluids have been developed to process polymer and lipid materials and produce particulate pharmaceuticals [107]. These techniques can be properly adapted to produce pharmaceutical grade protein delivery system formulations as they can avoid denaturation and degradation phenomena [108,109,110]. Recently, Salmaso *et al.* described a novel supercritical fluid gas micro-atomization process for the preparation of protein-loaded lipid particles [111]. They demonstrated that the gas micro-atomization process was suitable for the fabrication of lipid nanoparticles loaded with insulin and recombinant human growth hormone (rh-GH), two proteins of relevant pharmaceutical interest with significantly different physicochemical properties.

When using hot [112] or cold [113] HPH, the lipid is heated to approximately 5–10 °C above its melting point, then the drug is dissolved in the melt. For the hot homogenization technique, the drug-containing molten lipid is placed into a hot aqueous surfactant solution and stirred to obtain a good dispersion. The pre-emulsion is homogenized using a piston-gap homogenizer and the hot O/W nanoemulsion is then cooled down to room temperature, so that the lipid can crystallize again forming solid lipid nanoparticles. Crystallization can also be initiated at lower temperatures or by lyophilization. Cold homogenization technique is employed in the case of highly temperature-sensitive drugs or hydrophilic drugs. Both hot and cold HPH exclude the use of organic solvents, which could deactivate the drug or produce undesired effects in the body. The HPH equipment can affect the particle characteristics [114].

To prepare SLNs by the microemulsion technique [115], a mixture of water, surfactant (e.g., phospholipids) and co-surfactant (e.g., short-chain fatty acids) is heated to the lipid melting temperature and added under gentle stirring to the lipid melt. The compounds must be mixed in the correct ratio to provide a clear stable system for microemulsion formation: nanodrop diameter should be less than 150 nm. The microemulsion is then dispersed in a cold aqueous medium (2–3 °C) under mild mechanical mixing; the precipitated spherical particles have diameters of 70-200 nm.

Because of their lipid nature, SLNs are particularly well suited to load synthetic lipophilic drugs. Investigation of drug release kinetics and mechanism performed with etracaine, etomidate and prednisolone model drugs showed how this kind of carrier can be useful in the prolonged release of lipophilic drugs [116], while hydrophilic drugs would be partially lost during the hot homogenization process because of partitioning between the molten lipid and the water phase. SLNs prepared by hot HPH technique were loaded with tamoxifen, a nonsteroidal antiestrogen used in hormone-positive early breast cancer, and their antiproliferative activity was studied *in vitro* in the MCF-7 cell line. The resulted anti-tumor activity was comparable with that of the free drug, and the particle size was suitable for parenteral administration [117]. *In vivo* studies proved that SLNs stabilized with tristearin enhance the half-life and mean residence time in plasma of the anticancer drug tamoxifen citrate [118].

The use of SLN for the delivery of peptides and proteins (Table 1) was recently reviewed by Almeida *et al.* [119]. In one of the first studies concerning the incorporation of peptide-based species into lipid particles, SLNs were loaded with lysozyme, an enzyme capable of hydrolyzing 1,4-β-linkages in peptidoglycan and in chitodextrins; the protein maintained its activity during the process, thus proving that some proteins can endure the harsh procedures of formulation by HPH, making possible the use of SLN as antigen carriers for vaccine delivery [120]. Encapsulation of the model decapeptide gonadorelin in solid lipid nanoparticles prepared was performed by solvent diffusion in an aqueous system. The rather slow *in vitro* gonadorelin release proved the suitability of SLNs as a prolonged release formulation for hydrophilic peptide drugs [121]. SLNs were also evaluated as potential carriers for the delivery of recombinant yak interferon-α, which exhibits antiviral activity against vesicular stomatitis virus in Madin-Darby bovine kidney (MDBK) cells. SLNs with an average particle size of 124 nm were prepared by the double emulsion solvent evaporation (w/o/w) method. *In vitro* release study, antiviral activity measurements and cytotoxicity assays proved that interferon-loaded SLN could be a useful formulation for controlled release in veterinary therapeutics [122].

**Table 1:** Peptide and protein molecules incorporated in solid lipid nanoparticles.

Peptide/ Protein	Method of preparation	References
BSA	Adsorption onto SLN	[123]
Calcitonin	Solvent evaporation (w/o/w)	[124]
CyA	HPH hot dispersion	[96]
CyA	HPH cold dispersion	[125]
CyA	Warm microemulsion (o/w)	[126]
Gonadorelin	Solvent displacement	[55]
HSA	Adsorption onto SLN	[127]
Insulin	Solvent evaporation (w/o/w)	[128]
Insulin	Warm microemulsion	[129]
Insulin	Solvent displacement	[129]
Insulin	Supercritical CO_2_ (PGSS)	[130]
[D-Trp-6] LHRH	Warm microemulsion (w/o/w)	[131]
Lysozyme	HPH cold dispersion	[120]
Ovalbumin	Melt-dispersion (o/w)	[132]
Thymopentin	Warm microemulsion	[133]

Abbreviations: CyA – cyclosporine A; has – human serum albumin; HPH – high pressure homogenization; LHRH – luteinizing hormone-releasing hormone; PGSS – particles from gas saturated solution technique.

Non-stealth SLNs usually accumulate in liver Kupffer cells after intravenous injection. In the case of liver diseases (hepatitis, hepatic neoplasms, visceral leishmaniasis), this feature can be exploited as a targeting strategy, but in other cases passive targeting should be avoided. Because of their physicochemical properties (particle size, surface charge, hydrophobicity), SLNs are mostly recognized by macrophages [134]. Several modifications have been made to achieve long circulation times by avoiding RES uptake as discussed for stealth liposomes. SLNs surface can be modified by hydrophilic polymers or copolymers to make the particles stealth toward RES. PEG stearate modified SLNs showed reduced uptake by mouse macrophages after intraperitoneal injection, and the reduction was proportional to the PEG chain length [135].

SLNs are suitable nanocarriers for brain delivery. The brain takes up SLNs probably because of the surface adsorption of blood proteins such as apolipoproteins, which can favor the adherence to endothelial cells of the blood-brain barrier (BBB). This effect was studied for the trypanocidal drug diminazene formulated as a lipid-drug conjugate; the drug is hydrophilic and cannot cross the BBB, but the lipid-drug conjugate can cross the barrier thus reducing central nervous system infection of *Trypanosoma brucei* infected mice [124].

A nanoparticulate system, consisting of lipid nanoparticles coated with chitosan (CS), was developed for the oral administration of peptide drugs [136]. In particular, they investigated the nanoparticle ability to incorporate and deliver the model peptide salmon calcitonin (sCT). The results showed that a CS coating could be formed around the lipid nanoparticles by simple incubation in CS solution. In addition, sCT could be efficiently associated to the nanoparticles. Following an initial burst, the systems provided a continuous and slow release of the associated peptide.

Constantinides *et al.* [137] developed self-emulsifying water-in-oil microemulsions incorporating medium-chain glycerides. Formulation of calcein (a water-soluble marker molecule), or SK&F 106760 (a water-soluble RGD peptide) resulted in significant bioavailability enhancement in rats relative to their aqueous formulations.

### 3.4. Polymeric Nanoparticles

Polymeric nanoparticles are nanosized colloidal materials able to encapsulate, adsorb or covalently bind drugs. Since most polymer properties can be easily modified, nanoparticles constitute a versatile drug delivery system, which can be tailored to make the particles able to penetrate through biological barriers and to deliver drugs to cells or into intracellular compartments.

Only a limited number of polymers can be used for the formulation of nanoparticles designed to deliver drugs *in vivo* [138,139]. Indeed, a suitable polymer must be quickly eliminated from the body to allow repeated administrations while avoiding accumulation. The polymer itself and its degradation products must be non toxic and non immunogenic. Finally, the prepared nanoparticles should be endowed with suitable bulk properties to encapsulate the selected drug and tunable surface properties to modulate their *in vivo* fate [140]. A list of the most widely used polymers is presented in Table 2.

There exist several protocols to manufacture polymer nanospheres, encapsulating a wide variety of therapeutic biomolecules. At the laboratory scale, the protocol may be as simple as the emulsification of a concentrated aqueous solution of protein or the freeze-dried solid, in solvent, followed by secondary emulsion in aqueous continuous phase: water-in-oil-in-water (w/o/w) or solid-in-oil-in-water (s/o/w) double emulsion–solvent evaporation. However, this apparent simplicity is misleading, since there remains almost intractable problems of protein unfolding and degradation [141], relevant with respect to fabrication, storage and release.

**Table 2:** Most widely used polymers constituting nanoparticles designed as drug carriers. With kind permission from Springer Science+Business Media: *Pharmaceutical Research*, Methods for the Preparation and Manufacture of Polymeric Nanoparticles, *26*, **2009**, 1027, Christine Vauthier and Kawthar Bouchemal.

Material	Full name	Abbreviation or Commercial name*
Synthetic homopolymers	Polylactide	PLA
	Poly(lactide-*co*-glycolide)	PLGA
	Poly(ε-caprolactone)	PCL
	Poly(*iso*butylcyanoacrylate)	PICBA
	Poly(*iso*hexylcyanoacrylate)	PIHCA
	Poly(*n*-butylcyanoacrylate)	PBCA
	Polyacrylates and polymethacrylates	Eudragit*
Natural polymers	Chitosan	
	Alginate	
	Gelatin	
	Albumin	
Copolymers	Polylactide-poly(ethylene glycol)	PLA-PEG
	Poly(lactide-*co*-glycolide)-poly(ethylene glycol)	PLGA-PEG
	Poly(ε-caprolactone)-poly(ethylene glycol)	PCL-PEG
	Poly(hexadecylcyanoacrylate-co-poly(ethylene glycol) cyanoacrylate)	Poly(HDCA-PEGCA)
Colloid stabilizers	Dextran	
	Pluronic F68	F68
	Poly(vinyl alcohol)	PVA
	Copolymers (see above)	
	Tween® 20 or Tween® 80	

**Figure 3:**
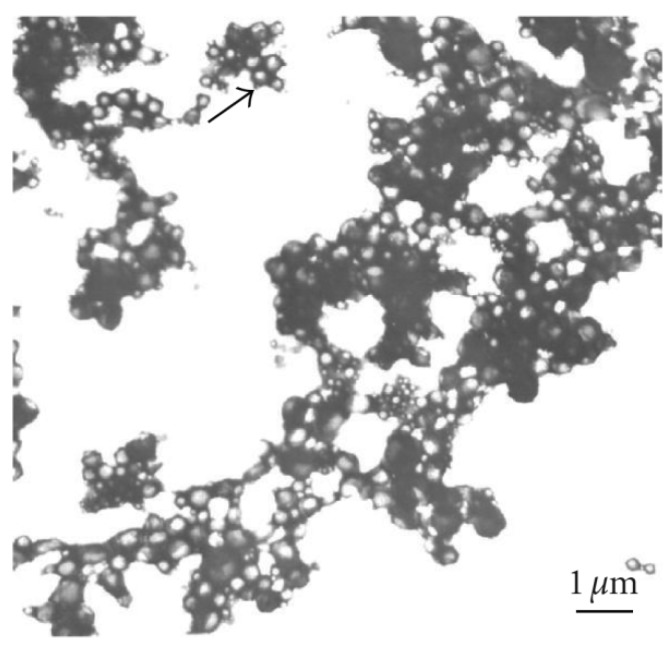
Transmission electron micrograph of chitosan-coated PBCA nanoparticles. The arrow points at a single nanoparticle. Reprinted with permission from ref. 143 under the terms of the Creative Commons Attribution license.

The selection of a proper polymeric carrier can also allow for sustained and controlled release of the drug. Moreover, a recent study showed how nanoparticle size can affect the biodistribution of targeted and non-targeted nanoparticles in an organ specific manner [142]. Many natural and/or synthetic polymers have been employed for the preparation of targeted protein nanocarriers. This collection includes gelatin, albumin, chitosan, blends of human serum albumin and the butyl hemiester of the alternating copolymer of maleic anhydride and 2-methoxyethyl vinyl ether, poly(L-lactic acid) (PLA), poly(lactic acid-*co*-glycolic acid) (PLGA), PEG-PLA block copolymers, blends of albumin and PLA, poly(*n*-butyl cyanoacrylate) (PBCA) and chitosan-coated PBCA (Figure 3), and poly(*n*-hexadecyl cyanoacrylate) [36,39,143,144,145]. Polystyrene, which is one of the most widespread polymeric materials, has recently been used as a model polymer for novel strategies in the field of drug delivery. For instance, polystyrene was used to prepare streptavidin-coated nanoparticles conjugated with a biotinylated monoclonal antibody against a surface protein of *L. monocytogenes* bacteria. After coupling with the bacteria, the conjugates were successfully used to deliver fluorescent or bioluminescent genes into cells [146]. Poly(L-lysine)-coated polystyrene nanoparticles were also used to enhance the efficacy of DNA vaccines [147]. However, polystyrene carriers can only be employed as model systems, since they are not biodegradable and thus cannot be cleared from circulation after therapeutic action.

Many methods have been developed or adapted for the preparation of polymer nanoparticles. Most of these methods include two main steps: preparation of an emulsified system followed by nanoparticles formation. The latter step can be achieved by polymer precipitation or gelation or by monomer polymerization. In general, the principle of this second step gives its name to the method. Some other methods do not require the first step and the nanoparticles are formed by polymer precipitation in conditions of spontaneous dispersion formation by macromolecule self-assembly to form nanogels or polyelectrolyte complexes. Table 3 summarizes the most common methods for nanoparticle preparation together with their main advantages and disadvantages. Updated information on the diversity of methods that can be applied to produce polymer nanoparticles was recently reviewed by Vauthier and Bouchemal [138].

Poly(*n*-butyl cyanoacrylate) nanoparticles were used to target neurons and neuronal cell lines. The particles were taken up by primary hippocampal cultures, thus delivering intact proteins inside the cells [148]. Surface modification of poly(*n*-butyl cyanoacrylate) nanoparticles with polysorbate 80 (a nonionic surfactant and emulsifier derived from polyethoxylated sorbitan oleate, commercially available under the trade name Tween 80) proved to cross the blood brain barrier *in vivo* [149]. Thanks to their high resistance and ability to deliver drugs to RES organs [150], other cyanoacrylic polymers such as poly(*n*-hexadecyl cyanoacrylate) [151] and poly(methoxypoly(ethylene glycol) cyanoacrylate-*co*-*n*-hexadecyl cyanoacrylate) [152,153,154,155] have been used for the preparation of nanoparticles.

**Table 3:** General advantages and disadvantages of the nanoparticle preparation methods. With kind permission from Springer Science+Business Media: *Pharmaceutical Research*, Methods for the Preparation and Manufacture of Polymeric Nanoparticles, *26*, **2009**, 1049, Christine Vauthier and Kawthar Bouchemal.

Method	Advantages	Disadvantages
By using a colloidal mill	Production of well characterized emulsions, uniform size. Easy to scale-up	High energy for the emulsification process
Emulsification–solvent evaporation	Possibility to encapsulate both hydrophilic and lipophilic drugs	Possible coalescence of the nanodroplets during the evaporation process
Emulsification–solvent diffusion	Control of nanoparticle size. Easy to scale-up	High volumes of water to be eliminated Leakage of water-soluble drug into the saturated-aqueous external phase
Emulsification–reverse salting-out	Minimal stress to fragile drugs. High loading efficiency. Easy to scale-up	Possible incompatibility between the salts and drugs. Purification needed to remove electrolytes
By gelation of emulsion droplets	Possibility to use natural macromolecules, hydrophilic and biocompatible	Limited to the encapsulation of hydrophilic drugs
Polymerization of alkyl cyanoacrylates	Easy method to obtain core-shell tuned nanoparticles. Control of nanoparticle size sby using surfactant	Possible reaction between the drug and CeVI in the case of radical emulsion polymerization. Purification needed
Interfacial polycondensation	Low concentrations of surfactants. Modulation of the nanocapsule thickness by varying the monomer concentration	Limited to the encapsulation of lipophilic drugs Purification needed
Nanoprecipitation of a polymer	Simple, fast and reproducible. Low concentrations of surfactants. Easy to scale-up	Low polymer concentration in the organic phase
Formation of polyelectrolyte complexes	Easy to achieve. According to the nature of the polyelectrolyte used in excess, either positively or negatively charged nanoparticles can be synthesized	Necessity to optimize the ratio between negatively and positively charged molecules
Formation of nanoparticles from neutral nanogels	Organic solvent free method. Controlled drug release	Not yet applicable to hydrophilic drugs
Methods based on ionic gelation	Organic solvent free method. Possibility to control drug release encapsulated in the nanoparticles upon the action of a pH or an ion concentration variation stimulus	Possible particle disintegration due to the weakness of the ionic interactions

Water-soluble polymers such as poly(ethylene glycol) or poly(*N*-vinyl-2-pyrrolidinone) are often chosen to increase the solubility of lipophilic drugs like anticancer drugs, since poor solubility in water can produce scarce adsorption upon oral administration or cause embolism in case of intravenous injection [156]. Curcumin, a natural yellow polyphenol that shows strong anti-cancer effect in many human cancer cell lines, did not find widespread application because of its scarce solubility in water, and consequent low bioavailability. Encapsulation of curcumin into amphiphilic copolymers of *N*-*iso*propylacrylamide, *N*-vinyl-2-pyrrolidinone and poly(ethylene glycol) monoacrylate led to fast dispersion in aqueous environment without affecting the therapeutic properties [157]. However, polymers can also be used in bioimaging [158] and for the delivery of water-soluble proteins in order to reduce their denaturation. For instance, polyethyleneimine was used to synthesize cell-permeable and biocompatible polymeric nanoparticles, properly functionalized in order to bind a caspase-specific near-infrared fluorescence probe, Cy5.5-Asp-Glu-Val-Asp. Such nanoparticles are auto-quenched, but in the presence of caspase proteases 3 and 7 they are readily cleaved and produce higher fluorescence, thus proving useful to visualize caspase-dependent apoptosis [159].

**Figure 4:**
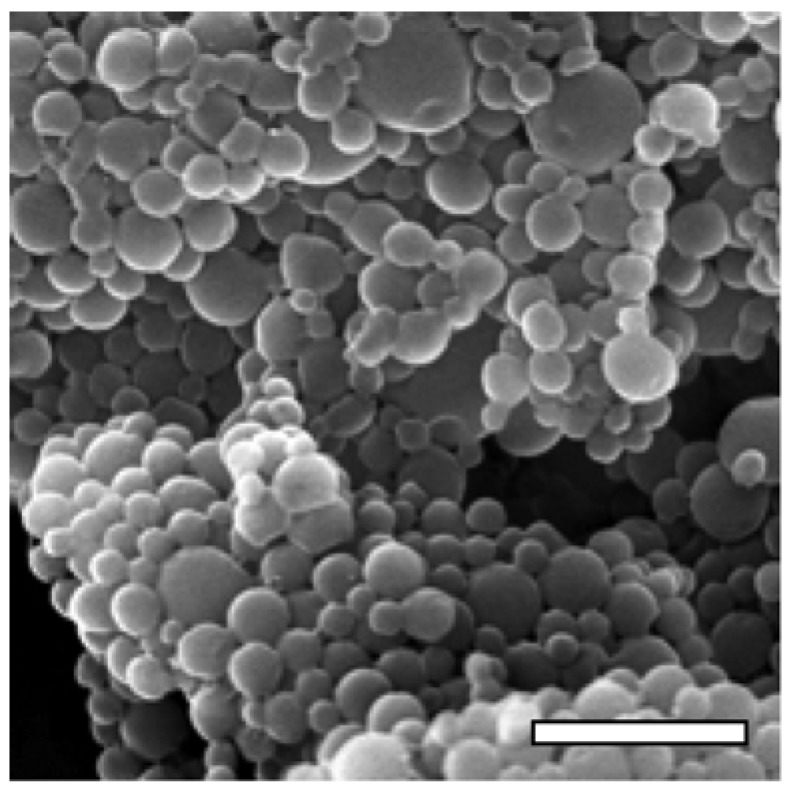
Scanning electron micrograph of PLGA nanoparticles produced in a contact-free flow-through ultrasonication cell (bar is 1 µm). Reprinted with permission from ref. [162].

Among the different polymers, particular attention has been dedicated to biodegradable polymers. After the introduction of bioresorbable sutures, the concept of a self-eliminating material was adapted to drug delivery. The use of such materials avoids the problem of surgically removing the exhausted delivery system after the administration, thus reducing toxic effects due to polymer accumulation and surgical complications because of the removal; typical examples are PLA and PLGA [160,161,162], which are often used for the preparation of biodegradable particles (Figure 4). In particular, Freitas *et al.* [162] presented a novel and elegant method to produce PLGA nanoparticles in a continuous, contact- and contamination-free process that can be readily run under sterile conditions. During the entire manufacturing process, the product is in direct contact only with sterile glass and Teflon® tubes. The process can be run in a closed system involving a contact-free flow-through ultrasonication cell in order to prevent any environmental contamination.

As polyesters in nature, these polymers undergo hydrolysis upon implantation in the body, forming biocompatible and metabolizable by-products (lactic acid and glycolic acid) that undergo the citric acid cycle and are removed from the organisms. Polymer biodegradation products are formed very slowly and do not affect the normal cell function. They can also protect the loaded drug against degradation while providing control over the release kinetics, which is proportional to the polymer decomposition rate. For instance, even if insulin cannot be normally administered orally because of degradation due to proteolytic enzymes in the gastrointestinal tract, poly(ε-caprolactone) (PCL) blends, PLA and PLGA were tested for the oral delivery of insulin or insulin derivatives to diabetic rats [163,164], and they showed that in some cases the loaded drug could successfully decrease glycemia. PEG-coated PLA nanoparticles and chitosan-coated PLGA nanoparticles loaded with insulin and tetanus toxoid for oral and nasal administration showed how the surface treatment with hydrophilic polymers could improve the stability of the particles and enhance the trans-mucosal protein transport and delivery [165]. Biodegradable PEG-PLGA block copolymers were used for the encapsulation of the enzyme catalase and provided 20% encapsulation efficiency and retained 25–30% of enzymatic activity for at least 18 hours in a proteolytic environment, while free catalase lost activity within one hour [166].

PLA-PEG nanoparticles have also been used as carriers for proteins. Tobio *et al.* attempted to explore the potential of these systems as transmucosal carriers for proteins [167]. With this idea in mind, they encapsulated radiolabeled tetanus toxoid into PLA and PLA-PEG nanoparticles and evaluated tetanus toxoid absorption following nasal and oral administration to rats. Irrespective of the administration route, the toxoid levels in the blood stream and lymph nodes were significantly higher for PLA-PEG nanoparticles than for PLA nanoparticles. In light of the results obtained in a later paper, Vila *et al.* [168] concluded that PLA-PEG nanoparticles work as carriers that are able to efficiently transport the encapsulated tetanus toxoid through the nasal mucosa. In addition, they concluded that the extent of the absorption of the toxoid encapsulated into the particles was dependent on the size of the particles, being more important for the nanoparticles than for the microparticles.

Simone *et al.* synthesized a series of PEG-PLA diblock copolymers containing about 14–93% PLA and having total molecular weight ranging from about 4 to 100 kDa [169,170]. Upon processing by the freeze-thaw double-emulsion solvent-evaporation technique, all diblock copolymers formed polymer nanocarriers, which encapsulated the potent antioxidant enzyme catalase. The encapsulated enzyme proved to be resistant to protease degradation. The PEG/PLA ratio affected the nanoparticle yield, shape, stability, loading, activity, and protease resistance of encapsulated catalase. The nanoparticles transitioned from spherical to filamentous shapes, increasing the hydrophobic polymer fraction from 14 to 93%, consistent with trends for self-assembly of lower molecular weight amphiphiles. Importantly, one diblock copolymer formed filamentous particles loaded with significant levels of protease-resistant enzyme, demonstrating for the first time encapsulation of an active therapeutic enzyme into filamentous carriers. Nanoparticle morphology also greatly influenced its degradation, offering a new means of controlled delivery. As expected, the f-PNC stiffness, length, and thickness increased with increasing copolymer molecular weight. Interestingly, heating above the polymer glass transition temperature (<30 °C) increased the f-PNC flexibility.

PLA and PLGA are not the only biodegradable materials employed for the preparation of nanoparticles for drug delivery. Copolymers of methacryloylglycylglycine and *N*-hydroxypropyl­methacrylamide were used for the preparation of HSA-loaded nanoparticles, with 60% encapsulation efficacy, a time-dependent release profile *in vitro* and high cytocompatibility [171]. Owing to their relative stability in extracellular medium and to their rapid degradation in intracellular environments, water-soluble polyamidoamines containing repetitive disulfide linkages can be used for the intracellular delivery of protein such as β-galactosidase [172]. Amphiphilic poly(amino acid) derivatives such as hydrophobically modified poly(γ-glutamic acid) can form nanoparticles in water; when loaded with ovalbumin or recombinant HIV, the particles are efficiently taken up by immature dendritic cells, thus proving suitable for vaccine delivery [173].

Other naturally occurring polymers, especially polysaccharides such as chitosan and alginate or combinations thereof, can be used as biodegradable materials for the preparation of nanoparticles. *N*-trimethylchitosan nanoparticles were evaluated both *in vitro* and *in vivo* for the delivery of a model protein (fluorescein isothiocyanate labeled bovine serum albumin) and a model vaccine (urease) and turned out to be suitable as potential protein/vaccine carriers for oral delivery [174]. Alginate/chitosan nanoparticles loaded with insulin proved to be very effective in oral dosage to diabetic rats, lowering basal serum glucose levels by more than 40% [175].

**Figure 5:**
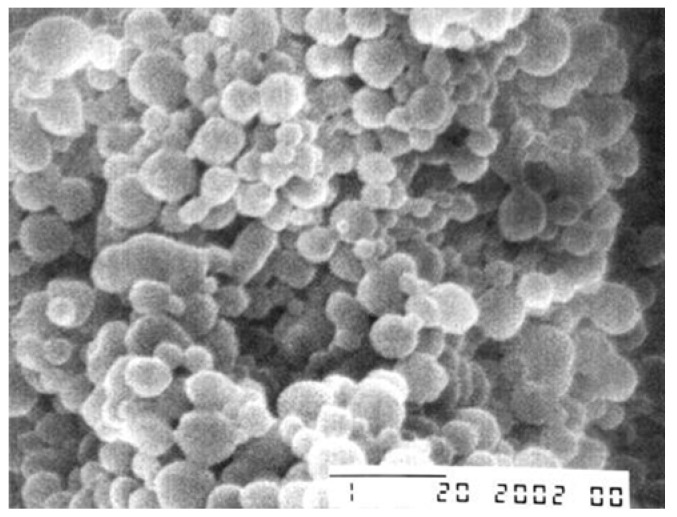
SEM micrograph of nanoparticles obtained by co-precipitation of the *n*-butyl hemiester of the alternating copolymer of maleic anhydride/tetraethylene glycol vinyl ether and HSA and coated with digalactosyl diacyl glycerol as targeting moiety (bar is 1 μm) [178].

Albumin particles can be loaded with anti-inflammatory drugs like celecoxib, an anti arthritic drug; *in vitro* studies showed that the particles sustained the release of the drug for about six days, and *in vivo* the accumulation of the radiolabeled drug loaded particles in the inflamed joint was 2.5-fold higher than in a non inflamed joint, while no significant difference could be seen for radiolabeled celecoxib [176]. Albumin can also be used in combination with synthetic polymers; hybrid nanoparticles composed of human serum albumin (HSA) and hemiesters of alternating copolymers of maleic anhydride/alkyl vinyl ethers of oligo(ethylene glycol) were prepared and made able to target liver hepatocytes thanks to digalactosyl diacyl glycerol (Figure 5), a natural glycolipid selectively recognized by the asialofetuin receptor [177,178]. The aforementioned alternating copolymers were also used for the formulation of bioerodible polymer matrices in conjunction with HSA and stabilized with β-cyclodextrins, providing good *in vitro* and *in vivo* biocompatibility [179].

As the commercialization of nanotechnology continues to expand, the ability to translate particle-fabrication methods from a laboratory to an industrial scale is of increasing significance. A very recent review examines several of the most readily scalable top-down methods for the fabrication of such shape-specific particles and compares their capabilities with respect to particle composition, size, shape, and complexity as well as the scalability of the method [180]. The authors offer an extensive examination of particle replication in non-wetting templates (PRINT) with regard to the versatility and scalability of this technique [181,182,183]. They also detail the specific methods used in PRINT particle fabrication, including harvesting, purification, and surface-modification techniques, with an examination of both past and current methods. For example, insulin, albumin, and albumin mixtures with siRNA or paclitaxel were molded using PRINT to yield monodispersed micro- and nano-sized protein particles. Nano-molding was achieved by a lamination technique where an aqueous protein solution was placed between a patterned PFPE mold and a polyethylene sheet. The PFPE mold, aqueous protein solution and polyethylene sandwich structure, were passed through a roller with an applied pressure of 50 psi. As the mold passed under the roller, the high-energy film was peeled away, leaving a filled mold of individual cavities containing the aqueous protein solution. The filled mold was subsequently frozen and lyophilized overnight to remove water.

Recently, an original method of producing aqueous-core lipid nanocapsule, able to encapsulate both hydrophilic and lipophilic species with relevant yields, was presented [184]. In fact, two fundamentally different kinds of nanocapsules are defined according to the nature of the materials constituting their liquid core, that is, either oil- or aqueous-core nanocapsules, both dispersed in a continuous water phase. Oil-core nanocapsules are the most widely encountered systems, owing to their easier formulation. However, these oil-core nanocapsules systems only prove useful for the encapsulation of lipophilic species. The proposed process comprises three steps: the formulation of water-in-oil nanoemulsions; *in situ* nanoemulsion interfacial polymer synthesis; removal of the continuous oil phase and addition of external water. As a proof of concept, tolylene 2,4-di*iso*cyanate was used as polymerizable monomer, whereas methylene blue and fluorescein-labeled BSA were selected as lipophilic and hydrophilic drug, respectively. The resulting colloidal objects presented a particular, stratified morphology and were able to encapsulate simultaneously, and with significant yields, hydrophilic molecules in the aqueous core and lipophilic molecules in the oil shell. Moreover, the adopted low-energy procedures should prevent the degradation of the fragile molecules to be encapsulated.

Owing to the insolubility of most organic polymers in water, nanoparticle preparation often requires the use of organic solvents that could cause protein denaturation and toxic effect. As a consequence, there has been a quest for more friendly approaches to polymeric nanoparticle formulation. In particular, there has been a growing interest in the development of drug carriers that are formed in water by self-aggregation of hydrophobically modified water-soluble polymers [185]. They consist of a hydrophilic polymer backbone, on which hydrophobic moieties are grafted. One of the most interesting features of these polymers is their unique associative behavior in aqueous solutions, due to the strong tendency of their hydrophobic groups to aggregate in order to minimize their contact with the solvent. The mode of association depends on polymer concentration, and on structural parameters such as the content, length and distribution of the hydrophobic groups along the polymer backbone. At sufficiently high polymer concentrations, intermolecular associations prevail and act as transient crosslinks connecting polymer chains. Among the associative polymers, hydrophobized polysaccharides are particularly attractive due to their biocompatibility, biodegradability and low toxicity, which are advantageous for biological and pharmaceutical applications. For instance, polysaccharides that can self-assemble to form nanoparticles and associate with various proteins were proposed as potential drug carriers [186,187]. Moreover, these nanocarriers are prepared in water in the absence of organic solvents, thus avoiding or at least limiting the denaturation of protein drugs.

In recent years, the supercritical antisolvent (SAS) precipitation technique has emerged as a promising “green” method for the formation of fine particles. Despite its numerous advantages, this technique still cannot easily produce soft particles smaller than 300 nm. An improved SAS process can however produce particles of controllable size, up to an order of magnitude smaller than those of the conventional SAS process, with a narrower size distribution [188]. The reliability of this process is demonstrated by the formation of lysozyme nanoparticles that retain the protein biological activity. An improved system using both supercritical antisolvent precipitation and rapid expansion from supercritical to aqueous solution (RESAS) was proposed to overcome the problem of low solubility of medicinal substances in supercritical CO_2_ [189]. On the other hand, supercritical CO_2_ methods cannot be easily applied to the production of polymer nanocarriers because of the insolubility of organic polymers in supercritical CO_2_ [190]. Accordingly, supercritical CO_2_ can only be used as antisolvent to form nanoparticles from polymer solution in an organic solvent.

### 3.5. Protein Conjugates

Despite the good results obtained using nanocarriers for drug delivery, it is also clear that in some cases even the small size of nanocarriers can limit the efficacy of cell specific receptors that should drive the drug to the desired cell. Since it is not possible to indefinitely reduce the size of the particles, a possible approach to avoid this drawback consists of eliminating the carrier. In this case, the protein drug must be conjugated with the targeting moiety able to selectively deliver the drug to the appropriate tissue [191]. Strictly speaking this approach is outside the scope of the present review. Nonetheless, protein conjugates cannot be completely ignored if taking into account the huge number of relevant publications. Accordingly, this section will only present a quick overview of their potential application.

Protein conjugates can be especially useful to specifically deliver proteins, enzymes, small molecules or DNA into mitochondria to treat mitochondrial diseases. Since mitochondria play an important role in apoptosis, the ability to deliver proteins such as superoxide dismutase (to protect mitochondrial DNA and nuclear DNA from reactive oxygen species), the apoptosis-inducing protein (for cancer therapy) and the anti-apoptosis protein (therapy for cardiomyopathy induced from excess apoptosis) into mitochondria can be of great importance. The most important strategies to deliver proteins into mitochondria consist of conjugating the protein to mitochondrial targeting signal peptides (MTS) or to protein transduction domains (PTD) [192]. MTS are short peptide sequences located at one end of the precursor protein, able to drive the protein into the mitochondrion. After entering the organelle, MTS are cleaved, thus releasing the fused protein and allowing its localization and functionality. In addition to the aforementioned proteins, this method can be used to deliver restriction enzymes able to digest mutant DNA deriving from mitochondrial disease producing specific elimination of the altered DNA [193].

PTD are specific domains of about 10–16 residues, that are able to cross and deliver cargos through biological membranes. In particular, arginine-rich peptides have the ability to penetrate cell membranes and to bring exogenous proteins into the cells [194]. A well-known example is the PTD from HIV-1 TAT protein, which consists of 11 amino acids including six arginine and two lysine residues [195]. Such domains can be fused with peptides, proteins or other species such as liposomes or low-molecular weight compounds, and penetrate barriers (even the blood-brain barrier) to deliver cargo molecules to the desired tissue both *in vivo* and *in vitro* [196]. PTD can reach the cytosol, but is not able to specifically target organelles. PTD and MTS can then be combined to achieve efficient cytoplasmic and mitochondrial protein delivery as shown by Shokolenko *et al.*, who prepared a fusion protein consisting in exonuclease III protein conjugated to TAT and MTS. The protein was successfully delivered into breast cancer cell mitochondria making the mitochondrial DNA prone to oxidative stress [197].

Several protein toxins in bacteria are composed of different domains that control different functions. One of the domains is responsible for the selective binding to target receptors; a second domain assures the permeation of the complex into the cytosol *via* receptor-mediated endocytosis; a third domain is the real toxin and can affect intracellular enzymatic processes leading to cell death [198]. Once the toxin has been removed or inactivated, the other domains can be exploited to deliver molecules inside cells. *Clostridium botulinum* neurotoxins are the most dangerous for humans. They target neurons and cause flaccid paralysis, muscle coordination disorders and breathing muscles paralysis, thus leading to death. However, once the active domain of the toxin has been removed, the rest can be used to selectively deliver proteins to neurons [199]. The specificity of botulinum binding domain for cholinergic nerve terminals can be exploited to improve the gene delivery by viral vectors to motor neurons in gene therapy of amyotrophic lateral sclerosis (ALS). The botulinum-binding domain can be fused with streptavidin thus making it possible to bind any biotinylated viral vector carrying the gene of interest and to deliver it to the motor nerve terminal [200]. Recently, was also demonstrated that genetic modification can be carried out on *Clostridium botulinum* toxin delivery domain to make it target non-neuronal cells in order to broaden its therapeutic field of application. The modified toxin was used to target and cleave SNAP23, a non-neuronal SNARE (Soluble *N*-ethylmaleimide-sensitive factor Attachment protein REceptors) protein that mediates vesicle-plasma membrane fusion processes involved in human hypersecretion diseases, thus proving the feasibility of its application in the treatment of similar pathologies [201]. If *Clostridium botulinum* neurotoxin provides an opportunity to dispense drugs to peripheral neurons, *Clostridium tetani* neurotoxin allows for the delivery to spinal cord in a similar way, and in general the clostridial neurotoxins can be recombined to optimize properties for clinical use, as recently reviewed [202].

The selection of peptide ligands for individual proteins, and thus to desired cells, can be carried out thanks to peptide libraries displayed on icosahedral T7 phage. This includes the identification of peptide ligands for receptors, such as integrins, cytokine and growth factor receptors. The selected peptides can then be used as targeting moieties. For example, the mutated tail fiber protein p17 expressed by 20–6 phage (a mutated phage) proved to be highly selective for hepatocyte targeting in rhesus monkeys. Fusion proteins prepared with either enhanced yellow fluorescent protein or α-interferon and injected into mouse tail vein accumulated mostly in hepatocytes [203]. After proper functionalization, this peptide was used to deliver dextran, small interfering RNA, liposomes and nucleic acid complexes to mouse hepatocytes [204].

**Figure 6:**
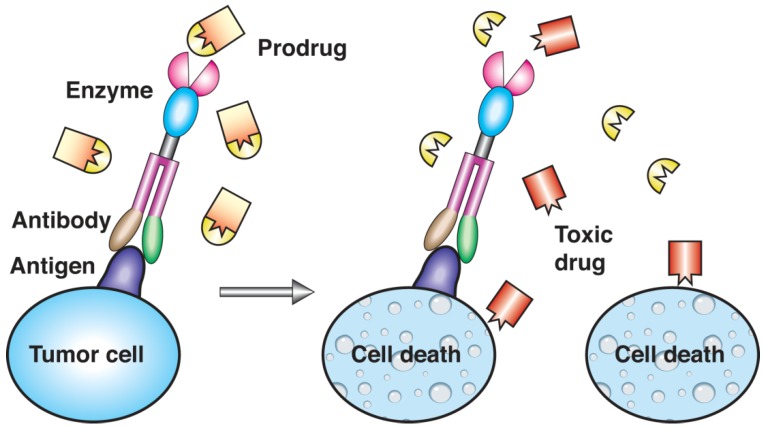
Schematic diagram of the principle of ADEPT. An antibody-enzyme fusion protein, injected i.v., is allowed to localize to tumors. After enzyme activity is cleared from circulation, a prodrug remains and the enzyme cleaves the prodrug to release the active drug. The extracellularly generated drug can diffuse throughout the tumor and kill antigen positive tumor cells as well as tumor cells not expressing the relevant antigen, thus giving a ‘‘bystander’’ effect. Adapted from ref. [205].

Antibody-directed enzyme prodrug therapy (ADEPT) represents a potentially very useful approach to cancer therapy, although no protein drug is actually delivered by this technique. ADEPT consists of selectively delivering an enzyme to tumor antigens, thanks to the conjugation to a specific antibody. After the accumulation of the conjugate in the desired tissue and clearance of the unbound conjugate from the blood stream, a non-toxic prodrug is injected and conversion of the prodrug into a cytotoxic drug occurs in the target tissue, where it meets the localized enzyme (Figure 6). No side effects should be caused elsewhere in the body. *In vivo* studies proved that a single ADEPT cycle performed using MFE-CP (a multifunctional genetic fusion protein of antibody and enzyme) can reproducibly reduce tumor growth rate in established human colon carcinoma xenografts, while multiple cycles can even cause regression. Mannosylation of the complex was performed in order to increase the clearing from normal tissues *via* hepatic mannose receptors, and this method significantly enhanced the simplifying of ADEPT strategies for human applications [205].

## 4. Targeting Strategies and Applications

### 4.1. Topical Application

The simplest method of driving a drug to a specific region within the body consists of locally applying the drug to the selected area. Even if this strategy cannot properly be assimilated to targeted delivery, it is at the borderline between systemic delivery and targeted delivery. Indeed, topical applications reach the goal to limit the area within which the drug can diffuse, thus avoiding most systemic side effects. When a drug is dispensed systemically, most of the product is wasted because of its diffusion through healthy organs, which can suffer damage, and only a small amount of the drug reaches the target tissue or organ. Direct application allows for better control over the amount of drug that reaches the area of interest; increasing the local concentration while reducing the drug diffusion within the rest of the body. This method proved to be successful in the delivery of liposome-entrapped drugs, *i.e.,* in the intra-articular administration of hormone-based drugs totreat arthritis [206] and in the intracoronary infusion of thrombolytic enzymes to cure myocardial infarction [207]. Non-encapsulated falintolol used as a beta-blocking agent has been successfully delivered by direct application in open-angle glaucoma therapy [208]. A recent feasibility study demonstrated how topical application of metformin could help bone regeneration around dental implants in type 2 diabetic patients [209].

**Figure 7:**
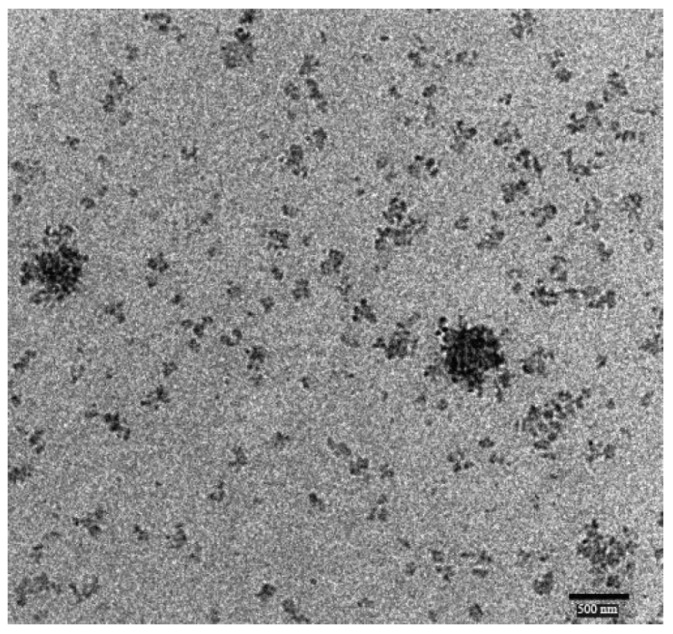
TEM image of flurbiprofen solid lipid nanoparticles. Reprinted from ref. [212] under the terms of the BioMed Central Open Access license.

In addition to the need for skilled personnel and often for hospitalization of the patient, local application is not specific and cannot be carried out if the target tissue is hard to reach or delocalized, or if the drug is not available in a liquid form in case of administration by syringe. In such cases, the drug may be loaded into a nanocarrier for delivery inside the body. Local application of solid lipid nanoparticles loaded with retinol and incorporated in an o/w emulsion can influence the drug penetration and release inside the skin layers [210]. Although in some cases the permeation of the drug through the skin can be enhanced by the use of nanocarriers [211], particles often have to be incorporated in pharmaceutical preparations like creams, lotions and ointments, and are mostly used for cosmetic purposes, but also for transdermal administration of cortisonic, anti-inflammatory or antibiotic drugs. Another possibility consists of incorporating the drug loaded nanocarrier into hydrogels; solid lipid nanoparticles and nanostructured lipid carriers loaded with flurbiprofen and dispersed in hydrogels (Figure 7) for transdermal delivery. This method proved to increase the *in vivo* bioavailability of the drug by 4.4-times that of oral administration [212].

### 4.2. Enhanced Permeability and Retention Effect

**Figure 8:**
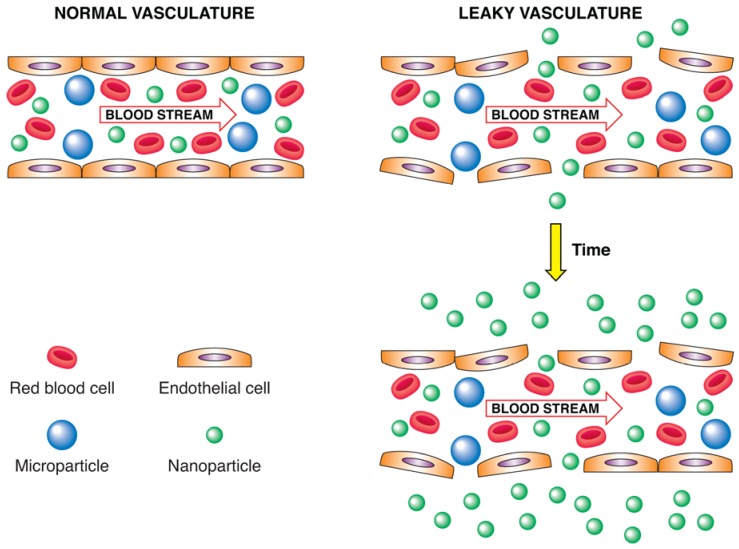
Illustration of the EPR effect. Only particles smaller than the cut-off size can permeate the vessel walls and extravasate to the tissues, eventually resulting in enhanced retention and accumulation of particles due to slow lymphatic clearance.

Blood vessels increase their permeability when affected by solid tumors or by inflammatory or infectious processes [213]. The vessels become leaky, thus allowing particles to cross the wall and permeate the interstitial space. The size of the particles can vary from 10−500 nm, thus including liposomes or micelles. The nature of the disease affects the porosity of the vasculature, allowing for control over diffusion of the drug; the choice of a properly sized carrier would allow the drug to extravasate from the blood vessel (Figure 8). Moreover, tumor cells lack an effective lymphatic drainage system. Both aspects facilitate structures with a size up to approximately 200 nm to accumulate in tumor tissue. Maeda and co-workers named this phenomenon “Enhanced Permeability and Retention (EPR) effect” and widely investigated this method as a targeting solution for cancer, especially when using macromolecular drugs [214,215,216,217,218,219,220].

To exploit this targeting method, drug carriers should circulate in blood long enough to provide acceptable accumulation of the active molecule in the area of interest. PEG-coated liposomes loaded with highly toxic anticancer drugs such as anthracyclines circulate for a long time in the blood flow, thus exerting a positive effect in cancer therapy with reduced side effects [221]. The PEG coating inhibits liposome uptake by the reticuloendothelial system and significantly extends liposome residence time in the bloodstream. Polyphosphazenes-platinum (II) conjugates afford high tumor selectivity by EPR effect,allowing for controlled release of the active platinum moiety, avoiding problems due to unfavorable pharmacokinetics and short circulation time of platinum-based anticancer drugs [222]. Mouse model studies showed how the EPR effect is responsible for the accumulation of PEGylated anti-inflammatory antibody in inflamed joints after intravenous administration [223].

### 4.3. Physical Targeting

The concept behind physical targeting consists of the targeted delivery of drugs mediated by physical alterations of the area of interest. This technique can be applied following one of two main approaches. The first one relies on the intrinsic properties of the injured area; indeed, inflamed or neoplastic areas differ from normal tissues, often showing higher temperature or lower pH. In such conditions, it is possible to employ drug carriers able to survive in normal tissues, but subjected to degradation at lower pH or higher temperatures; in this case drugs are only released in the injured area, without undesired systemic effects. The second approach consists of the application of an external stimulus, like heat or a magnetic field, to degrade the carrier and release the drug. When the application is strictly localized, the drug accumulates only inside the area of interest.

Nanoparticles can be engineered to obtain the required properties. Cholesterol-grafted poly(*N*-*iso*propylacrylamide-*co*-*N*,*N*-dimethylacrylamide-*co*-undecenoic acid) can be coupled with folate to make it able to target folate receptors overexpressing cancer cells. Nanomicelles of about 200 nm can then be prepared by membrane dialysis method, in order to exploit the hydrophobic core to encapsulate hydrophobic anticancer drugs like paclitaxel or doxorubicin. *In vitro* cytotoxicity assays performed against KB cells evidenced an enhanced cellular uptake of micelles surface-functionalized with folate [224] due to a receptor-assisted endocytosis process. It was demonstrated *in vitro* that this kind of nanoparticle shows pH-induced thermosensitivity, which is a useful feature to induce drug release only inside the cells and not in the extracellular environment [224,225].

**Figure 9:**
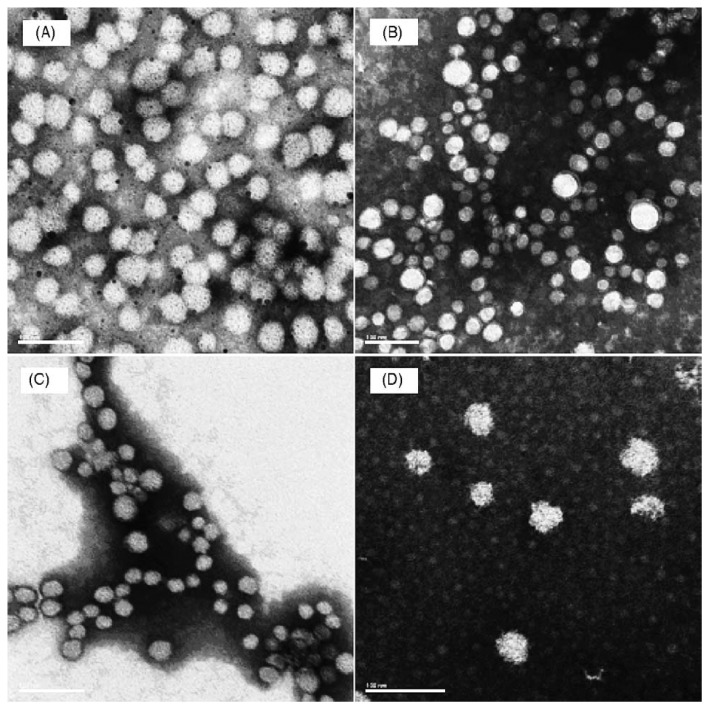
TEM micrographs of CyA-pH sensitive nanoparticles prepared by using (a) poly(*N,N*-dimethylaminoethyl methacrylate-*co*-butyl methacrylate-*co*-methyl meth­acrylate) (Eudragit E100); (b) poly(methacrylic acid-co-ethyl methacrylate) 1:1 (Eudragit L100-55); (c) poly(methacrylic acid-*co*-methyl methacrylate) 1:1 (Eudragit L100); (d) poly(methacrylic acid-*co*-methyl methacrylate) 2:1 (Eudragit S100). Reprinted with permission from ref. 226, Copyright © 2009, Elsevier B.V.

pH-sensitive nanoparticles can also be used for the oral delivery of cyclosporine A (CyA), a cyclic endecapeptide frequently used as immunosuppressant after transplantation surgery. The possibility to release the drug at a specific pH allows for delivery within the gastrointestinal tract, increasing the probability of drug absorption, which is usually low for oral delivery, while decreasing the degradation by gastric acid and gastrointestinal enzymes. To this purpose, CyA was loaded in pH-sensitive nanoparticles made of ionic methacrylic copolymers [226], such as poly(*N,N*-dimethylaminoethyl methacrylate-*co*-butyl methacrylate-*co*-methyl methacrylate) (Eudragit E100), poly(methacrylic acid-*co*-ethyl methacrylate) 1:1 (Eudragit L100-55), and poly(methacrylic acid-*co*-methyl methacrylate) 1:1 (Eudragit L100) and 2:1 (Eudragit S100) (Figure 9). CyA-loaded nanoparticles (37–107 nm average diameter) were prepared by using an adaptation of the quasi-emulsion solvent diffusion technique [227]. *In vitro* release experiments revealed that the nanoparticles exhibited perfect pH-dependant release profiles. The relative bioavailability of CyA markedly increased for S100, L100-55 and L100 nanoparticles and decreased for E100 nanoparticles when compared with the commercial Neoral microemulsion. “Smart” pH-sensitive polymers can turn from hydrophilic to hydrophobic inside the endosome, thus destabilizing the endosomal membrane and causing carrier release. Such polymers can then deliver therapeutic peptide, protein and nucleic acid molecules past the endosomal membrane into the cytoplasm of targeted cells [228].

An efficient method to target drugs to specific areas consists of applying an external magnetic field. The drug must be loaded or immobilized on ferromagnetic nanocarriers, able to respond to magnetic stimuli, and then injected intravenously. The localized magnetic field induces the accumulation of the particles in the area of interest. This idea dates back to 1960, when Freeman *et al.* proposed that magnetic nanoparticles could be delivered inside specific areas of the body thanks to a magnetic field [229]. The particles can be both organic (polymeric) and inorganic or composite materials, and in addition to drug delivery they can also be used for imaging techniques such as MRI and can undergo heating in magnetic fields to induce hyperthermia of tissues. Contrast agents for MRI are frequently made from super-paramagnetic iron oxide nanoparticles (SPION), which show excellent magnetic properties and low toxicity for the body. They can be coated with non-polymeric [230] or polymeric [231,232] materials to improve their performances. Recent reviews focus on technical aspects of magnetic targeting as well as nanoparticle design and animal and clinical trials [233,234].

**Figure 10:**
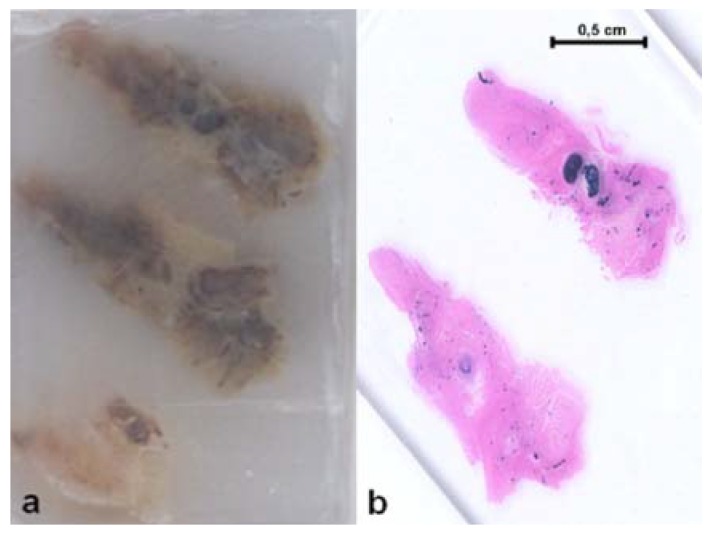
(a) VX2-tumour tissue after magnetic drug targeting embedded in paraffin. (b) Synoptic picture of the corresponding histological slide stained with Prussian blue. The nanoparticles are visible as dark spots in the tumor vessels. Reprinted with permission from ref. [237].

*In vivo* studies performed on rabbits with induced VX-2 squamous cell carcinoma using starch-coated magnetic iron oxide nanoparticles bearing mitoxantrone, an anticancer drug, proved how magnetic targeting of the drug can efficiently remove the tumor using smaller amounts of mitoxantrone than in conventional intra-arterial application of the drug [235]. The application method can influence the response to the treatment: intravenous administration of the nanoparticles was not effective, while the intra-arterial application gave good results despite the potential thrombotic risk (Figure 10) [236,237].

Because of the intrinsic limitations of magnetic targeting, therapeutic applications of magnetic nanoparticles are mostly limited to delivery of anticancer drugs. Some examples of protein loaded magnetic nanoparticles are however reported in the literature. Dextran coated SPION nanoparticles functionalized with streptokinase, a thrombolytic enzyme, were injected into the arteries while an external magnet was applied over the thrombus [238]. Although the dosage of streptokinase was lower than generally used in clinics, all thrombi could be dissolved in contrast to the control groups where no external magnetic field was applied. Immobilization of BSA on SPION nanoparticles was performed by two different double-step immobilization approaches [239].

The first approach consists of preparation of SPION by controlled chemical coprecipitation in the presence of BSA solution, whereas the second approach includes preliminary surface modification of SPION with 3-aminopropyltriethoxysilane (APTES). Both procedures are followed by carbodiimide with sequential immobilization of the layer of BSA. BSA-coated SPION retained the superparamagnetic properties while reducing the value of saturation magnetization (Ms). APTES-coated SPION showed higher BSA binding capacity compared to that of coprecipitated SPION in the presence of BSA. BSA-coated SPION incubated *in vitro* with human dermal fibroblasts cells demonstrated a cell response similar to that of control cells, with no adverse cell damage and no endocytosis. Fe_3_O_4_ nanoparticles conjugated to proteins were developed in order to potentially serve as protein carriers [240]. The surface of the nanoparticle was first modified with PEG, then APTES was added to form a self-assembled layer. Nanoparticles were finally conjugated with lysozyme as a model protein by amidation with glutaraldehyde. The strategy was relatively straightforward for simultaneous synthesis of particles on a nano scale and polymeric surface modification. A trifluoroethylester-terminal PEG silane was synthesized and self-assembled on iron oxide nanoparticles [241]. The resulting nanoparticle system could be conjugate with cell targeting agents *via* either carboxylic or amine terminal groups for a number of biomedical applications, including magnetic resonance imaging and controlled drug delivery. The conjugating flexibility was demonstrated with folic acid. TEM analysis showed the presence of well-dispersed nanoparticles before and after they were coated with PEG and folic acid.

The different features offered by magnetic particles can also be combined in multi-responsive nanocarriers. One example consists of the use of polypeptide-based diblock copolymers poly(butadiene-*b*-glutamic acid) combined with hydrophobically modified γ-Fe_2_O_3_ nanoparticles. These superparamagnetic hybrid nanocarriers can be deformed by magnetic fields, and at the same time they are sensitive to stimuli such as pH and ionic strength, due to the presence of the polypeptide block [242].

### 4.4. Molecular Targeting

The use of targeting moieties is an active strategy that relies on specific interactions at target sites; such interactions include antibody-antigen and ligand-receptor interactions. The “magic bullet” initially theorized by Paul Ehrlich has evolved in a three-parts system composed of a therapeutic agent, a carrier for the drug and a targeting moiety combined together. The targeting moiety has to be specific for the area of interest, thus making the distribution of the drug independent from the EPR effect. This targeting strategy provides evident advantages such as high specificity for the injured area and reduced side effects, but it can show its best performances in cancer therapy, especially for the cure of delocalized tumors or tumors in their early stages of development, when the vasculature is still immature. Potential targeting moieties include antibodies and their fragments [243], aptamers (protein binding DNA) [244,245,246], peptides [247,248], proteins such as transferrin [249,250] or lectins [251], saccharides [252], hormones [253], glycoproteins [254] and vitamins, especially folate [255].

In spite of the potential benefits of targeted nanocarriers, these systems have some drawbacks such as the cost and stability of the targeting moiety. To justify the increased cost, the moiety must significantly increase the efficacy of the nanovector. Peptides and aptamers are often more stable than carbohydrates or antibodies [51], they still can undergo proteolysis *in vivo*; they may lose their targeting ability or elicit an immune response. Similarly, nuclease activity can degrade aptamers [244]. Another concern is that the targeting ligand itself could elicit an immunogenic response in a patient, although this issue is more prominent for antibodies [256]. One further drawback of targeted delivery is the effect the uptake pathway may have on these systems. Targeted liposomes are often taken up and transported to the harsh environment of lysosomes [256]. Another problem of targeted drug delivery is the depth of penetration into the target tissue. It has been reported that targeted liposomes bind to the first few cell layers after extravasation from the vasculature and retard the entry of following liposomes [257]. This phenomenon is correlated with the size of the nanovector and binding affinity of the targeting ligand; bigger nanovectors and stronger binding ligands penetrate shorter distances [258]. Despite many of these still standing challenges, targeted nanocarriers represent a promising strategy for future development of targeted delivery therapeutics.

Antibodies are highly selective for the relevant antigen, and this feature can be exploited for precise delivery of drugs to desired tissues. Moreover, it is now possible to create monoclonal antibodies, *i.e.,* antibodies designed to be specific for almost any substance, obtained from a single clone of an immune cell. They can be engineered in several ways in order to meet specific requirements from different biological environments [259]. Antibodies are proteins composed of IgG, which contains an antigen-binding fragment (Fab, responsible for specific antigen binding) and a complement-fixing fragment (Fc, responsible for fixing complement for *in vivo* biological response). Recombinant antibody technology allows for the preparation of a library of antibodies from which the ones with the required properties can be selected.

Monoclonal antibodies can be used to deliver peptide radiopharmaceuticals through the BBB for imaging brain tumors, especially during their early stages when the BBB is still intact. Imaging is also useful to detect extracellular amyloid in order to monitor other neurological disorders like Alzheimer. The small size of these short radiolabeled peptides provide fast blood clearance and suitable pharmacokinetics, and different peptide sequences, both natural or synthetic, can be labeled with iodine, technetium, indium, gallium, carbon or fluorine [260]. The 83–14 monoclonal antibody to the human insulin receptor, tagged with streptavidin, was used to deliver the biotinylated ^125^I-Aβ^1-40^ (^125^I-labeled 40-residue β-amyloid peptide) to the brain of rhesus monkeys, showing good uptake of the radiolabel from the brain and a 90% clearance after 48 hours [261].

Cystatin, a protein inhibitor of cysteine proteases with potential antitumoral activity, was incorporated in PLGA nanoparticles [262], which were further surface-modified with a monoclonal antibody recognizing a specific antigen overexpressed in invasive breast tumor cells (MCF-10A neoT). *In vitro* tests showed that the immunonanoparticles were able to recognize and target the antigen on MCF-10A neoT cells in a co-culture with other cells. Following endocytosis, cystatin delivered by immunonanoparticles effectively inhibited intracellular cathepsin B only in the target cells.

Polymeric nanoparticles displaying tumor necrosis factor on their surface are useful carrier systems capable of mimicking the bioactivity of membrane-bound TNF leading to a striking enhancement of apoptosis. However, potential systemic toxicity hampers their *in vivo* application. To overcome this issue, Messerschmidt *et al.* [263] investigated the possibility to combine the advantages of polymeric nanoparticles and liposomes for the generation of multifunctional lipid-nanoparticle composite systems in which the TNF activity is shielded. As a model system, they employed polystyrene-based nanoparticles with a single-chain TNF-functionalized surface, which were coated with a sterically stabilized PEG-lipid shell, further endowed with a targeting moiety by insertion of single-chain Fv-PEG-lipids into the lipid coat. Antibody fragments lacking the Fc-region, e.g., Fab′ or single-chain Fv (scFv), can be used to avoid recognition by Fc receptor-bearing cells of the RES [264]. As target antigen they used fibroblast activation protein (FAP), a cell surface dipeptidase overexpressed by tumor stromal fibroblasts. *In vitro* the targeted nanocarriers specifically bound to FAP-expressing, but not to FAP-negative cells. Lipid coating strongly reduced nonspecific binding of particles and scTNF-mediated cytotoxicity towards FAP-negative cells. In contrast, the nanocarriers showed an increased cytotoxicity for FAP-positive cells.

Bionanocapsules (BNCs) are nanoparticles with a high biocompatibility composed of the L protein of the hepatitis B virus surface antigen [265] that can efficiently and specifically deliver bioactive molecules to hepatocytes. However, delivery is limited to hepatocytes and incorporation of proteins into BNC is quite troublesome. In order to alter the specificity of BNC and to achieve efficient protein delivery, Kurata *et al.* [266] developed engineered BNC displaying the ZZ domain of protein A and incorporating enhanced green fluorescent protein (EGFP) as model protein drug. The ZZ domain displayed on the surface of BNC binds to antiepidermal growth factor receptor (EGFR) antibodies, allowing specific delivery of EGFP to HeLa cells. According to the authors, engineered BNCs are a promising and powerful tool for efficient and cell-specific protein delivery.

PLGA nanocarriers targeted to intercellular adhesion molecule-1 (ICAM-1) were evaluated for the delivery of acid sphingomyelinase (ASM), an endothelial surface protein upregulated in many pathologies. This approach is well suited for lysosomal diseases, which manifest by peripheral organ dysfunction. This is the case for type B Niemann-Pick disease, caused by a genetic deficiency of acid sphingomyelinase (ASM), which leads to aberrant accumulation of sphingomyelin and cholesterol [267]. Real-time vascular imaging using intravital microscopy and postmortem imaging of mouse organs showed rapid, uniform, and efficient binding of fluorescently labeled nanocarriers to endothelium after i.v. injection in mice [268]. Fluorescence microscopy of lung alveoli actin, tissue histology, and ^125^I-albumin blood-to-lung transport showed that anti-ICAM nanocarriers cause neither detectable lung injury, nor abnormal vascular permeability in animals. Radioisotope tracing showed rapid disappearance from the circulation and enhanced accumulation of anti-ICAM/^125^I-ASM nanocarriers in kidney, heart, liver, spleen, and primarily lung. These data demonstrate that ICAM-1-targeted nanocarriers may enhance enzyme replacement therapy for type B Niemann-Pick disease and perhaps other lysosomal storage disorders.

ICAM-1 is a good target for targeting nanoparticles to the perturbed endothelium. Coupling ICAM-1 antibodies to nanoparticles created multivalent ligands that enter cells *via* an endocytic pathway [269]. Internalized anti-ICAM nanoparticles were retained in a stable form in early endosomes for an unusually long time, and were subsequently degraded following slow transport to lysosomes. Inhibition of lysosome acidification delayed degradation without affecting anti-ICAM trafficking, whereas the microtubule disrupting agent nocodazole retarded degradation by inhibiting anti-ICAM nanoparticle trafficking to lysosomes. Endothelial cells were resistant to H_2_O_2_-induced oxidative injury after uptake of catalase-loaded nanoparticles. Chloroquine and nocodazole increased the duration of antioxidant protection by decreasing the extent of anti-ICAM/catalase degradation. The concomitant ICAM-1 disappearance from the endothelial cell surface transiently inhibited subsequent binding and uptake of anti-ICAM nanocarriers [270]. Within one hour, internalized ICAM-1 resurfaced, and enabled uptake of a subsequent anti-ICAM/nanoparticle dose. Thus, internalized ICAM-1 was able to recycle back to the plasma membrane. *In vivo* pulmonary targeting of anti-ICAM nanoparticles affected neither EC viability nor endocytosis and traffic to lysosomes. However, lysosomal trafficking of the second dose of anti-ICAM nanoparticles was decelerated, suggesting that recurrent targeting to ICAM-1 affords longer effects. *In vitro* and *in vivo* investigation of the targeting to endothelial cells by anti-ICAM PLGA nanoparticles carrying approximately 200 anti-ICAM molecules demonstrated that the nanoparticles quickly and specifically bound to tumor necrosis factor-activated endothelial cells and bound to endothelial cells and accumulated in the pulmonary vasculature after i.v. injection in mice [271]. Anti-ICAM nanoparticles displayed markedly higher endothelial cell affinity than naked anti-ICAM in cell culture. These results demonstrate that reformatting monomolecular anti-ICAM into high-affinity multivalent nanoparticles boosts their vascular immuno-targeting. As indicated, targeting nanocarriers loaded with antioxidant enzymes (e.g., catalase) to endothelial cell adhesion molecules alleviates oxidative stress in the pulmonary vasculature. However, antioxidant protection is transient, since catalase is internalized, delivered to lysosomes, and degraded. Manipulation of the cellular elements controlling the uptake and intracellular trafficking of anti-ICAM nanoparticles allowed for modulating the metabolism and longevity of endothelial cell-targeted drugs and hence the duration of the therapeutic effect [272]. Antibody-directed targeting of nanocarriers to platelet–endothelial cell adhesion molecule PECAM-1, an endothelial glycoprotein containing six Ig-like extracellular domains, was recently investigated by the same research group [273]. PECAM-1 antibodies bind to endothelial cells without internalization, but endothelial cells internalize by endocytosis nanocarriers carrying multiple copies of anti-PECAM. To determine whether binding and intracellular transport of anti-PECAM nanoparticles depend on the epitope engaged, we targeted five PECAM-1 epitopes. *In vitro* experiments demonstrated that endothelial binding, endocytosis, and intracellular transport of anti-PECAM nanocarriers are epitope-specific.

Aptamers proved to be useful in the *in vitro* selective delivery of highly toxic *cis*platin-based drugs to prostate cancer cells by incorporation into A10 prostate-specific membrane antigen targeted nanoparticles of PLA-PEG-COOH [274]. The same strategy was used *in vitro* to deliver PLA-PEG-COOH nanoparticles loaded with rhodamine-labeled dextran to prostate LNCaP epithelial cells [246].

Specific peptides can also be used to target nanocarriers. The peptide KRTGQYKLC, which is recognized by fibroblast growth factor (FGF) receptors by binding to basic FGF, can be used for drug delivery to tumor cells overexpressing FGF receptors. Further functionalization with 1,2-distearoyl-phosphatidylethanolamine-methyl-polyethyleneglycol conjugate (mPEG-DSPE) reduces most of the interaction with erythrocytes and macrophages, prolonging blood circulation time of peptide grafted PEGylated liposomes [275].

Kogure *et al.* [276] developed a multifunctional envelope-type nano device (MEND) capable of cytoplasmic delivery of nucleic acids. The MEND is composed of a condensed core of DNA with polycations, which is covered with lipid membranes. A MEND that is covered with a high density of octaarginine can stimulate macropinocytosis and is taken up efficiently [277]. Suzuki *et al.* [278] applied the MEND to protein delivery. A model protein, GFP, was condensed with stearyl octaarginine (stearyl R8) to form nanoparticles that were then coated with a lipid membrane, thus permitting R8 to be introduced for efficient cellular uptake and controlled intracellular trafficking. The packaging efficiency of the MEND was significantly higher than that of conventional liposomes. Moreover, the MEND was internalized efficiently and effectively escaped from the acidic compartment to release GFP into the cytosol. These results indicate that the MEND can serve as a useful cytoplasmic delivery system for protein therapy.

Demirgöz *et al.* [279] designed and assessed peptide functionalized polymer vesicles, or polymersomes, self assembled from poly(ethylene oxide-*b*-butadiene) diblock copolymers for the treatment of prostate cancer. PR_b, a highly effective α_5_β_1_ targeting peptide that mimics the cell-adhesion binding-site in fibronectin, was conjugated onto the carrier surface with controlled orientation. PR_b-functionalized polymer vesicles effectively internalized within prostate cancer cells after adhering specifically to α_5_β_1_ integrins expressed on the cell surface. Nanoparticle internalization was found to depend on the surface concentration of PR_b. Different polymer vesicles encapsulating a model protein drug, tumor necrosis factor-α (TNFα), were tested *in vitro* on LNCaP human prostate cancer cells. PR_b-functionalized nanocarriers presented a dramatic improvement in the cytotoxic potential of the delivered TNFα, whereas polymer vesicles functionalized with the peptide GRGDSP did not show improvement with respect to non-functionalized polymer vesicles.

It is now known that several peptides and proteins can cross the cellular membrane in a process named protein transduction, thus delivering and releasing loaded molecules into the cytosol. These species are named protein transduction domains or cell-penetrating peptides and have been used for intracellular delivery of several high molecular weight molecules [280]. TAT and other cell penetration peptides can be exploited to make polymers, nanoparticles and liposomes able to efficiently penetrate cells [281]. Transferrin is a suitable targeting moiety that can be coupled to liposomes in order to make them able to selectively deliver the drug to tumor cells that overexpress transferrin receptors. Liposomes loaded with horseradish peroxidase and tagged with transferrin proved to be able to deliver the drug to brain capillary endothelial cells *in vitro* [50], thus becoming suitable drug carriers for brain delivery. Mixed strategies can also be applied: labeling PEG-coated liposomes with OX26 monoclonal antibody to the rat transferrin receptor allowed for the delivery of anticancer drugs to the rat brain through the blood-brain barrier [282]. Receptor-mediated endocytosis can even be used to deliver protein conjugates to cancer cells without the need for a carrier [283].

Lectins belong to a class of proteins characterized by the ability to bind carbohydrates with high specificity. Therefore, they can be exploited to target nanocarriers at glycosylated cells, such as cells from human gastrointestinal tract. As an alternative to injections, oral administration of wheat germ agglutinin-modified SLNs loaded with insulin enhanced the intestinal absorption of insulin sufficiently enough to drop the glucose level in blood [129]. Even if insulin is a hydrophilic peptide, it can be incorporated with high efficiency (about 98%) into SLNs showing good physical stability and sustained drug release behavior [284]. On the other hand, oligosaccharide ligands can be used to target cell-surface lectins [285], as well as selectins [286] and immunoglobulins [287]. Cyclodextrin-modified liposomes equipped with adamantoyl saccharides showed enhanced ability to cross the BBB endothelial cells *in vitro* [288].

Polymeric, dendritic and liposome nanocarriers can be directed specifically to tumor cells by adding a targeting moiety such as a synthetic analog of luteinizing hormone-releasing hormone. This peptide is a ligand for the receptors that are overexpressed in the plasma membrane of breast, ovarian, and prostate cancer cells and in some kinds of lung cancer cells, while healthy organs do not significantly express such receptors [289].

Endothelial-targeted biodegradable nanoparticles loaded with enzymes were prepared by double emulsion, freeze-thaw method [290]. Catalase, peroxidase and xanthine oxidase were loaded into 300 nm nanoparticles composed of biotin-terminated PEG-PLGA block copolymers. An antibody directed against platelet-endothelial cell adhesion molecule-1 (PECAM-1) was used to target endothelial cells. The antibody was conjugated to streptavidin and then bound to the nanoparticle surface. The enzyme-loaded nanocarrier were then tested both *in vitro* and *in vivo* to verify if this approach protects the endothelium against vascular oxidative stress, a pathological process implicated in ischemia–reperfusion and other disease conditions. Encapsulated catalase and peroxidase were protected from external proteolysis and exerted enzymatic activity on their nanoparticle diffusible substrates, H_2_O_2_ and *o*-phenylendiamine, whereas the activity of encapsulated XO was negligible due to polymer impermeability to the substrate (hypoxanthine). The reported results indicate that catalase-loaded nanoparticles targeted to PECAM-1 can protect the endothelium against oxidative stress in cell culture and animals.

Folate is a stable, non-immunogenic, easily available compound that can be used in organic solvents. The folate receptor is a glycosylphosphatidylinositol-linked membrane glycoprotein that is usually absent in normal tissues (except for placenta, choroids plexus and, in lower amounts, in lungs, thyroid and kidney), whereas it is frequently found in tumor tissues, and more than 90% of ovarian carcinomas overexpress folate receptor, thus making folate highly selective for ovarian carcinoma. Folate can be used to form conjugates with anticancer drugs like tubulysins, thus making them able to target cancer cells without the need for a carrier. Tubulysins are mixed nonribosomal-polyketide tetrapeptides that, in addition to isoleucine, contain three unique amino acids: *N*-methylpipecolinic acid, tubuvaline, and tubuphenylalanine. A semisynthetic analog of the microtubule inhibitor tubulysin B conjugated with folate showed remarkable antitumor activity *in vitro* and *in vivo* [291]. However, preparing drug conjugates is not always possible, because reactions on biologically active molecules can alter their functionality; in such cases, the use of carriers is more adequate. Nanoparticles prepared from *N*-trimethylchitosan, targeted with folate and loaded with fluoresceinated BSA showed that the intracellular uptake by SKOV3 cells (folate receptor overexpressing cells) was 3.7-fold more than that of untargeted particles, thus proving promising as protein carriers [292].

## 5. Concluding Remarks

The ability to deliver protein within the body is gaining paramount importance. Indeed, new molecules obtained thanks to bioengineering, e.g., hormones, vaccines, monoclonal antibodies, are leading to important changes in the therapy of acute and chronic diseases including cancer, infections and viral or autoimmune pathologies. The survival of such drugs in the body environment is difficult, due to the ease of protein denaturation or degradation unless a suitable carrier is used. The selected carrier must guarantee proper drug protection, be able to escape macrophage uptake, and its dimension should be tailored according to the specific needs. For different applications, it is necessary to formulate a proper carrier in terms of size, composition, surface functionalization, drug compatibility, targeting properties, thus making the process expensive and hardly scalable for industry.

Liposomes and solid lipid nanoparticles show high biocompatibility thanks to their lipid nature, and have the ability to encapsulate drugs and to release cargo in a controlled manner. On the other hand, they usually show considerable instability resulting in unsatisfactory shelf life and short circulation half time. Fusion proteins and virosomes can provide highly specific targeting, although they are easy marks for the immune system owing to their intrinsic immunogenicity.

Polymeric nanoparticles are endowed with a wide range of possible modifications, since their properties can be easily tailored to meet specific requirements. High loading efficiency is combined with the ease of surface functionalization; particle formulation and size are customizable, and cell targeting can be obtained by conjugation with a large number of targeting moieties. In addition, the uptake by the RES can be significantly reduced by proper surface modification. However, organic solvents are often used in the preparation of polymer nanoparticles and this could lead to toxicity and protein denaturation. These issues could be solved or at least mitigated by the adoption of more “green” methods of particle preparation, such as the use of self-assembling water-soluble polymers.

None of the reported nanocarriers can be considered the best choice for all potential applications. At present, however, polymeric nanoparticles seem to be the most versatile carrier for the targeted delivery of protein drugs. Indeed, the huge number of possible polymer structures and the large variety of preparation techniques allow for tuning the nanoparticle delivery system to the specific therapeutic application, administration route and type of protein drug.
